# NDRG1 and its family members: More than just metastasis suppressor proteins and targets of thiosemicarbazones

**DOI:** 10.1016/j.jbc.2025.110230

**Published:** 2025-05-14

**Authors:** Mahan Gholam Azad, Tiffany M. Russell, Xuanling Gu, Xiao Zhao, Vera Richardson, Tharushi P. Wijesinghe, Golap Babu, Xinnong Guo, Busra Kaya, Mahendiran Dharmasivam, Zhao Deng, Des R. Richardson

**Affiliations:** 1 Centre for Cancer Cell Biology and Drug Discovery, Institute for Biomedicine and Glycomics, Griffith University, Queensland, Australia; 2 Department of Pathology and Biological Responses, Nagoya University Graduate School of Medicine, Nagoya, Japan

**Keywords:** NDRG1, metastasis, metastasis suppression, thiosemicarbazone

## Abstract

N-Myc downstream regulated gene-1 (NDRG1) and the other three members of this family (NDRG2, 3, and 4) play various functional roles in the cellular stress response, differentiation, migration, and development. These proteins are involved in regulating key signaling proteins and pathways that are often dysregulated in cancer, such as EGFR, PI3K/AKT, c-Met, and the Wnt pathway. NDRG1 is the primary, well-examined member of the NDRG family and is generally characterized as a metastasis suppressor that inhibits the first step in metastasis, the epithelial–mesenchymal transition. While NDRG1 is well-studied, emerging evidence suggests NDRG2, NDRG3, and NDRG4 also play significant roles in modulating oncogenic signaling and cellular homeostasis. NDRG family members are regulated by multiple mechanisms, including transcriptional control by hypoxia-inducible factors, p53, and Myc, as well as post-translational modifications such as phosphorylation, ubiquitination, and acetylation. Pharmacological targeting of the NDRG family is a therapeutic strategy against cancer. For instance, di-2-pyridylketone 4,4-dimethyl-3-thiosemicarbazone (Dp44mT) and di-2-pyridylketone-4-cyclohexyl-4-methyl-3-thiosemicarbazone (DpC) have been extensively shown to upregulate NDRG1 expression, leading to metastasis suppression and inhibition of tumor growth in multiple cancer models. Similarly, targeting NDRG2 demonstrates its pro-apoptotic and anti-proliferative effects, particularly in glioblastoma and colorectal cancer. This review provides a comprehensive analysis of the structural features, regulatory mechanisms, and biological functions of the NDRG family and their roles in cancer and neurodegenerative diseases. Additionally, NDRG1-4 are explored as therapeutic targets in oncology, focusing on recent advances in anti-cancer agents that induce the expression of these proteins. Implications for future research and clinical applications are also discussed.

The process involved in the spread of cancer cells from the primary tumor to distal sites in the body is known as metastasis. In fact, metastasis is the major killer in cancer, resulting in 90% of deaths (1). Understanding the biochemical mechanisms that inhibit metastasis and deciphering molecular targets that can be pharmacologically modulated to block tumor spread are critical aims. Currently, there are no drugs used in the clinics that specifically target and inhibit metastasis (2, 3). In this regard, N-Myc downstream regulated gene-1 (NDRG1) is of interest as it was described in multiple cancers as a metastasis suppressor that inhibits multiple pro-oncogenic signaling pathways (4, 5, 6, 7, 8) and can be upregulated by pharmacopeia of the thiosemicarbazone class (9, 10, 11, 12, 13, 14).

NDRG1 was first identified as *calcium-associated protein 43* (*Cap43*), a gene upregulated in A549, human lung adenocarcinoma epithelial cells, in a dose- and time-dependent manner in response to non-toxic levels of nickel compounds using differential display technique (15). Subsequent sequence homology analysis led to the discovery of NDRG2-4 as closely related family members (16, 17). There have also been reports of NDRG1 playing a potential pro-oncogenic role in esophageal cancer (18), breast cancer (19, 20, 21), osteosarcoma (22), bladder cancer (23), hepatocellular cancer (24, 25, 26), gastric cancer (27), and non-small cell lung cancer (28). These paradoxical findings emphasize the importance of comprehensively understanding the biology of NDRG1 and its family members. A considerable body of research has begun to decipher the functions of NDRG1, which appears diverse and pleiotropic, making this protein an intriguing subject for investigation (29). Furthermore, NDRG1 is part of a family of four proteins, NDRG1, NDRG2, NDRG3, and NDRG4, which share 53% to 65% sequence identity (30), which could play separate or associated roles. Considering this, the current review will discuss the broad range of functions of NDRG1 and its family members, including their roles in disease and potential for cancer treatment.

While initially identified in mammals, NDRG family members are conserved across many metazoan species (31). Phylogenetic tree analysis has clearly demonstrated separate homology clusters for each corresponding NDRG family member, with around >80% sequence similarity detected between the species for individual NDRG family members (31). Additionally, within each family member, the evolutionary relationships follow expected taxonomic patterns, with humans and macaques clustering closely, followed by other mammals (*e.g.*, cow, mouse, rat), and then more distantly related vertebrates such as *Xenopus tropicalis* and *Danio rerio* from separate branches (31). In fact, these observations may support the fact that modern NDRG family members arose through multiple gene duplication events during evolution, with each paralogue retaining specialized functions. The NDRG2 lineage appears more closely related to the ancestral parent protein, YDJ1, which is derived from *Caenorhabditis elegans* (31). These conserved yet divergent features underscore the importance of understanding functional specialization within the NDRG family. Considering these factors, the current review will discuss the broad range of functions of NDRG1 and its family members. Further, phylogenetic analysis of the NDRG family suggests that NDRG1 and 3 belong to one protein subfamily, while NDRG2 and 4 belong to another (30).

### General roles and functions of NDRG1-4

Studies have revealed different roles and functions of the NDRG family members, with these ranging from cancer to placentation and endocrine signaling (31). Both *N-Myc* and *c-Myc* belong to a family of proto-oncogenes that encode transcription factors, which are often dysregulated in cancer (32). Of relevance to this, the expression of NDRG1 and NDRG2 has been demonstrated to be negatively regulated by both N-Myc and c-Myc expression, which play important roles in cellular proliferation (33, 34, 35). In contrast, NDRG3 and NDRG4 expressions were not demonstrated to have similar relationships with Myc proteins (31, 36).

The expression of NDRG1 was shown to be involved in many cellular processes, including the stress response, lipid biosynthesis, glycolytic and lipid metabolism, exocytosis, and differentiation (8, 37, 38). NDRG2 was found to play a role in cellular proliferation (39), astrocyte morphology (39), gliotransmission *i.e.*, the *Ca*^*2+*^*-dependent release of neurotransmitters such as glutamate, D-serine, and ATP by* astrocytes (40), and the formation of neuronal structures (41). Both *NDRG1* and *NDRG2* are considered to be hypoxia-responsive genes (42), while NDRG3 and NDRG4 function as neuroprotective proteins with roles that may potentially complement the upregulation of other neuroprotective molecules during hypoxia (43, 44, 45). These other neuroprotective molecules include vasoactive intestinal peptide (VIP) and pituitary adenylyl cyclase-activating peptide (PACAP) in rats (46). Research using zebrafish indicates NDRG4 has a role in signal transduction in myelinated axons (47). Moreover, NDRG4 was demonstrated to have functions in neuronal differentiation (48), maintenance of brain-derived neurotrophic factor (BDNF) (49), and is also involved in p53-mediated apoptosis after ischemic injury in rats (50).

Since NDRG family members play a role in neuronal function, these proteins could also be involved in neurological diseases, such as Alzheimer’s disease (44, 51, 52) and Parkinson’s disease (53, 54), as discussed further. Furthermore, the NDRG family was demonstrated to play important pro- and anti-oncogenic roles in cancer development (Table 1), including tumors such as meningioma, neuroblastoma, and glioma (44). Briefly, *NDRG2* was elucidated to be a tumor suppressor gene in meningioma, while NDRG4 has a proto-oncogenic role in this cancer (44, 55, 56). In aggressive childhood cancer, neuroblastoma, NDRG1 is considered to be a tumor suppressor, with its low expression being significantly associated with prognostic factors such as primary tumor size, *MYCN* amplification, and poor prognosis (57).

**Table 1:** The reported diverse roles of the NDRG family of proteins in a number of cancers

Name	Cancer-type	Expression levels	Function	References
NDRG1	Breast cancer (BC)	Subtype dependent	• Metastasis suppressor in hormone receptor-positive BC • Tumor promotor in aggressive and inflammatory BC	(7, 19, 38, 205, 302)
	Neuroblastoma		• Promotes multidrug resistance. • Inhibits PI3K/AKT signaling.	(251, 303)
	Pancreatic cancer	Low expression	• Inhibits WNT/β-catenin and PI3K/AKT signaling • Modulates chemoresistance	(103, 210, 282, 304)
	Hepatocellular carcinoma	Subtype dependent	• Promotes growth *via* preventing β-catenin degradation • Poor prognosis in hepatocellular carcinoma through mediating immune infiltration and EMT	(25)
	Prostate cancer	Low expression	• Inhibits androgen receptor signaling, inhibits cell proliferation, and induces apoptosis • Inhibits the epithelial-mesenchymal transition	(111, 208, 305)
NDRG2	Breast cancer	Low expression	• Inhibits JAK/STATs signaling • Suppresses cell migration *via* inhibition of NF-κB signaling	(216, 306)
	Neuroblastoma	Low expression	• Inhibits cellular proliferation	(217)
	Pancreatic cancer	Low expression	• Inhibits cellular proliferation	(218)
NDRG3	Invasive breast cancer, gastric cancer	High expression	• Related to poor patient survival • Related to imatinib resistance	(219, 220, 222)
NDRG4	Breast cancer, pancreatic cancer		• Tumor suppressor	(103, 223)

### Molecular structure of NDRG family members

Human NDRG family members share a 53% to 65% sequence identity between each family member, which is much lower in the *N-* and *C*-terminal regions (58, 59). Key structural features and similarities between family members are shown in Figure 1, *A–D*. Human NDRG1 contains 4 domains that could be responsible for different biological functions (31) (Fig. 1*A*). These domains include a phosphopantetheine attachment site (PPAS; amino acid 128–143), an NDR-domain, the α/β hydrolase domain (containing a cap-like region), and the 3 × 10 amino acid repeat (GTRSRSHTSE) (25). The cap-like region of NDRG1, which is also found in NDRG2-3 (Fig. 1*D*), has been demonstrated to be vital for both the stability and the activity of NDRG1. The 3 × 10 amino acid repeat (GTRSRSHTSE) within the *C*-terminal region of the NDRG1 is not unique to human NDRG1, as sequence homology analysis using BLAST revealed that the motif is highly conserved in NDRG1 orthologs in several species. Notably, this repeat is absent from other NDRG family members (Fig. 1*A*), which underscores its specificity to NDRG1 (59). Similarly, the PPAS is unique to NDRG1, and it is conserved in several metazoan species. However, it does not represent a canonical motif commonly found in other acyl carrier proteins. Given the structural resemblance of this motif to other phosphopantetheine attachment domains involved in lipid metabolism, this region may contribute to NDRG1’s specialized role in lipid-associated processes. Supporting this, a yeast two-hybrid screening indicated that NDRG1 has been linked to increased high-density lipoprotein-cholesterol (HDL-C) levels *via* interacting with HDLs and apolipoprotein A-I and A-II, suggesting a link between NDRG1 and lipid homeostasis, particularly in myelin-producing Schwann cells (60, 61).

**Figure 1:**
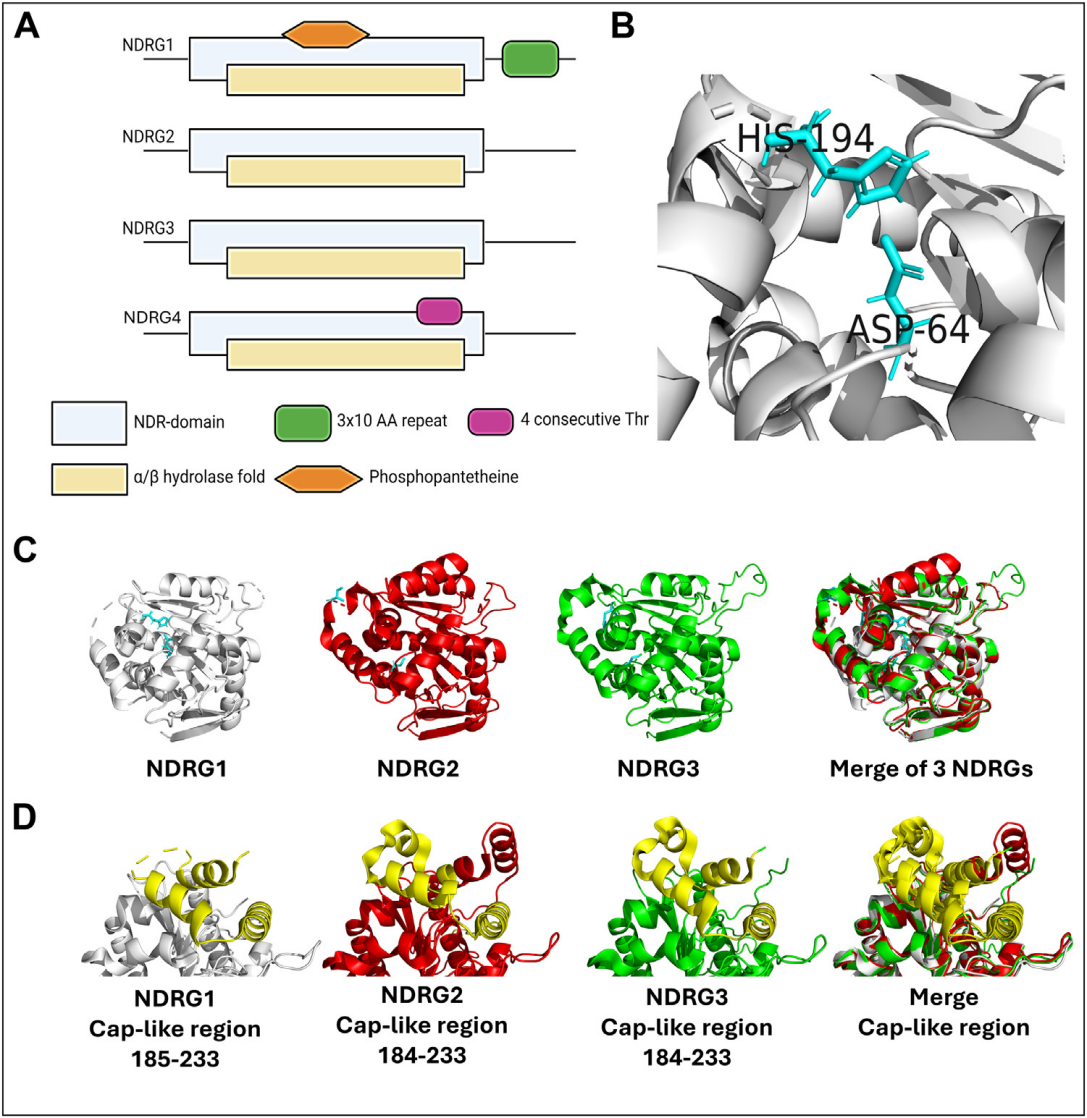
**Structure of NDRG family members.***A*, schematic of the overall structure of the NDRG1, 2, 3, and 4 proteins and the domains they contain (31, 59). The domains are: the NDR domain is a conserved region found within NDRG proteins, specifically consisting of a central α/β hydrolase-like region; a 3 × 10 amino acid (AA) domain (GTRSRSHTSE); a four consecutive threonine (Thr) domain; an α/β hydrolase domain; and a phosphopantetheine attachment binding domain (phosphopantetheine). Schematic modified from references (31, 59). *B*, the unique Asp64 and His194 catalytic site as part of the predicted α/β hydrolase region is only observed in NDRG1 and not other NDRG1 family members (59). *C*, the overall structure of NDRG1-3 demonstrates their similarity. *D*, comparison of the conserved Cap-like region in the NDRG1-3 proteins. *B–D*, were prepared using PyMOL (Schrödinger, LLC).

NDRG1 has also been suggested to interact with metal ions directly through its *C*-terminal region, which contains a 30-amino acid peptide, with the metal ions involved including nickel (62), copper (62), zinc (63), manganese, and cobalt (64). However, these latter studies were performed only using peptide fragments of NDRG1, and no physiologically relevant studies were performed to investigate whether the full-length protein could play a role in cellular metal ion metabolism. As such, the biological significance of this metal ion-binding property remains unclear and deserves further investigation.

Computational structural studies conducted by Shaw and colleagues (65) suggest that the NDRG proteins may be part of the α/β-hydrolase superfamily. This proposal is supported by the strong conservation of the α/β hydrolase domain between the NDRG family and the identification of the α/β hydrolase fold within each protein (65) (Fig. 1*A*). This α/β-hydrolase class of proteins is primarily composed of enzymes responsible for catalyzing a diverse range of reactions (65). Typically, the structural features of a α/β hydrolase family include: (1) an α/β hydrolase domain containing at least five parallel β-strands; (2) a catalytic triad arranged in the specific order nucleophile-acid-histidine; and (3) a nucleophilic elbow (65). However, the NDRG family members are unique in that they do not contain this typical catalytic triad observed in α/β hydrolases (65).

Although NDRG1 possesses no catalytic activity in terms of a hydrolase role, a catalytic (Asp64 and His194) site has been indicated in the α/β hydrolase region of NDRG1 (Fig. 1*B*), which is not observed in other NDRG family members (59). This Asp/His site is very similar to that observed in the *Bacillus subtilis* stress response regulator, RsbQ, which is an enzyme with hydrolase activity (59), with NDRG1 also being associated with the cellular stress response (31, 66). While NDRG1’s catalytic site may indicate potential hydrolase activity, there is no evidence to suggest it is functional. Examination of the α/β hydrolase domain within the crystal structures of human NDRG2 and 3 reveals the absence of the canonical catalytic triad and the potential blockage of a substrate-binding cavity by helices α7 and α10 (58, 67).

While human NDRG3 shares similarity to NDRG2 regarding its amino acid sequence and structure, the crystal structure of NDRG3 demonstrated considerable structural differences in a flexible loop analogous to helix α6 of NDRG2 that is thought to be involved in tumor suppression (67). It was suggested by these authors that this flexible loop region may play a role in the oncogenic progression induced by NDRG3.

NDRG4 contains a very unique threonine-rich region composed of 4 consecutive threonine residues (TTTT) near its *C*-terminal domain (68). Sequence homology analysis using BLAST indicates that this unique threonine-rich region is conserved across metazoan species, suggesting a functional adaptation of this motif in this paralog, which remains to be investigated further (68).

### Post-translational modifications of NDRG family members

Most knowledge regarding the post-translational modifications of NDRG family members is focused on NDRG1. Utilizing a *C-*terminus-directed antibody specific to the epitope at amino acids Gly382-Cys394, two distinct isoforms of NDRG1 were identified, namely a truncated version (∼41 kDa) and a full-length isoform (∼46 kDa) (69). While the mechanism behind the truncation of NDRG1 remains unclear, it is speculated that it may result from the proteolytic cleavage of the NDRG1 *N*-terminal or a distinct spliced variant (70). Notably, this truncated version is detected specifically in prostate cancer cells and not in mortal prostate epithelial cells (70), which suggests this NDRG1 truncation event may have a pathological relevance and could impact NDRG1 interactions and cellular distribution. Furthermore, it can be speculated that the levels of truncated NDRG1 compared to full-length NDRG1 could potentially affect its functional roles and suggest new avenues for future investigation.

In terms of post-translational modifications apart from cleavage (70), glycosylated forms of NDRG1 have not been observed, as treatment with a glycosidase did not alter the molecular weight of the 46 kDa isoform (70). However, human NDRG1 possesses phosphorylation sites at Thr3, Pro28, Ser330, Thr346, Thr356, and Thr366 (71, 72), and these can be regulated by serum/glucocorticoid-induced kinase 1 (SGK1). Phosphorylation by 101 primes NDRG1 for further phosphorylation of the serine residues by glycogen synthase kinase-3 (GSK3) at Ser342, Ser352, and Ser362 (72).

The *pro-viral integration site for Moloney murine leukemia virus 1 (PIM1)* is a proto-oncogene in several tumors, including breast, myeloid leukemia, prostate, and pancreatic cancer (73, 74, 75, 76), and encodes a serine/threonine kinase (77). PIM1 is transcriptionally upregulated by the Janus kinase 3 (JAK3)/signal transducer and activator of transcription 3 (STAT3) pathway and hypoxia (78, 79). PIM1 has been identified to regulate NDRG1 *via* phosphorylation at Ser330, resulting in a reduction in: (1) NDRG1 protein stability; (2) NDRG1 nuclear localization; and (3) the interaction of NDRG1 with the androgen receptor (AR); to enhance prostate cancer cell migration and invasion (80). This latter study highlights that the NDRG1 phosphorylation at Ser330 may be associated with higher-grade prostate tumors. Considering the importance of this observation, the role of PIM1 in the regulation of metastasis has also been linked to the interleukin-6 (IL-6)-induced epithelial mesenchymal transition (EMT) and stemness in breast cancer (81).

Studies have indicated that the levels of the Ser330 and Thr346 phosphorylated forms of NDRG1 were variably identified in six cancer cell types *in vitro*, namely, DU-145, PC-3, PANC-1, HT-29, HepG2, and Hep3B (69). Pronounced nuclear localization of p-NDRG1 (Ser330) was observed by Park *et al.*, which contrasts with the findings of Ladet *et al.* (62), who reported reduced nuclear localization of NDRG1 following Ser330 phosphorylation in LNCaP and VCaP prostate cancer cells. Phosphorylation of NDRG1 at Ser330 was demonstrated to be most pronounced in HT-29 colorectal cancer cells and PC3 prostate adenocarcinoma cells (69), while NDRG1 phosphorylation at Thr346 was predominantly observed in PC-3 cells and HepG2 hepatocarcinoma cells (69). Phosphorylation of NDRG1 at Ser330 and Thr346 has also been demonstrated to suppress CXC chemokines and nuclear factor kappa-light-chain-enhancer of activated B (NF-κB) activity (82). Hence, post-translational modifications and subcellular localization of NDRG1 may explain the pleiotropic functions of this protein.

Another approach to investigate the pleiotropic effects of NDRG1 was utilized to determine interactions with key proteins in prostate cancer and hepatocellular carcinoma cells (69), where NDRG1 has been elucidated to act in an anti- and pro-oncogenic manner, respectively (4). Considering that NDRG1 interacts with multiple proteins and signaling pathways, among these molecules, phosphatase and tensin homolog deleted on chromosome 10 (PTEN) was investigated in terms of its effects on NDRG1 (69). The latter protein was investigated since: (1) NDRG1 overexpression increases PTEN protein levels in prostate and pancreatic cancer cells (12, 83); (2) PTEN positively upregulates NDRG1 protein expression in prostate and breast cancer cells (84); and (3) PTEN is a tumor suppressor that dephosphorylates phosphatidylinositol-3,4,5-trisphosphate (PIP3) to phosphatidylinositol-4,5-bisphosphate (PI(4,5)P_2_), inhibiting the phosphoinositide 3-kinase (PI3K)/protein kinase B (AKT) pathway (85, 86).

*PTEN*-silencing resulted in increased levels of phosphorylated NDRG1 at Thr346 in DU-145 prostate cancer cells (69), where the anti-oncogenic effect of NDRG1 is observed (11, 87). In contrast, the levels of Thr346 phosphorylation did not change in HepG2 and Hep3B hepatocellular carcinoma cells (69), where a pro-oncogenic effect of NDRG1 was evident (26, 88). These data suggest that NDRG1 may have a cell-type-dependent interaction with PTEN, which may explain the mechanism for the pleiotropic activity of NDRG1 (69).

NDRG1 is post-translationally modified by the small ubiquitin-like modifier (SUMO) 2 protein, SUMO-2, at lysine 14, resulting in NDRG1 destabilization (89). As a result of SUMO-2 modification, the induction of p21 by NDRG1 was also inhibited (89), suggesting a functional role. Of note, NDRG2 also undergoes RNF4-mediated SUMOylation at Lys333 by a ubiquitin-like protein, SUMO1, in human lung adenocarcinoma cells (90). This latter effect results in the proteasomal degradation of NDRG2 to potentially promote lung cancer tumorigenesis (90).

Other post-translational modifications of human NDRG2 include phosphorylation of Thr330, Ser332, Thr348 (72), and Ser350 (91), which are regulated by SGK1, while Thr348 and Ser332 can also be phosphorylated by AKT and PKCθ in mice, respectively (92). Burchfield and colleagues have suggested that Ser332 phosphorylation by PKCθ may prevent insulin-induced Thr348 phosphorylation by AKT (92), which is relevant for the study of PKC-mediated insulin resistance. Phosphorylation of NDRG2 at Ser350 is mediated by death-associated protein kinase 1 (DAPK1), which has been linked to neuronal cell death in multiple neurological diseases such as Alzheimer’s disease (91).

Post-translational modification of NDRG3 and NDRG4 has not been extensively studied. Analysis of phosphoproteomics using the PhosphoSitePlus database (93) that integrates both low- and high-throughput data sources was used to examine post-translational modifications of NDRG1, 2, 3, and 4 (Fig. 2). Based on these data, the phosphorylation sites for all NDRG members are clustered at the *C*-terminal, with the ubiquitination sites at both the *N* and *C*-termini. Only NDRG1 and NDRG2 contain sumoylation sites at Lys14 and Arg247, respectively. Post-translational modifications are notably sparse at the *N*-terminal regions of all NDRG family members, except NDRG2, where there are seven phosphorylation sites, two acetylation sites, and one ubiquitination site (Fig. 2). These findings reveal possible novel regulatory mechanisms for NDRG1-4 that warrant further investigation.

**Figure 2:**
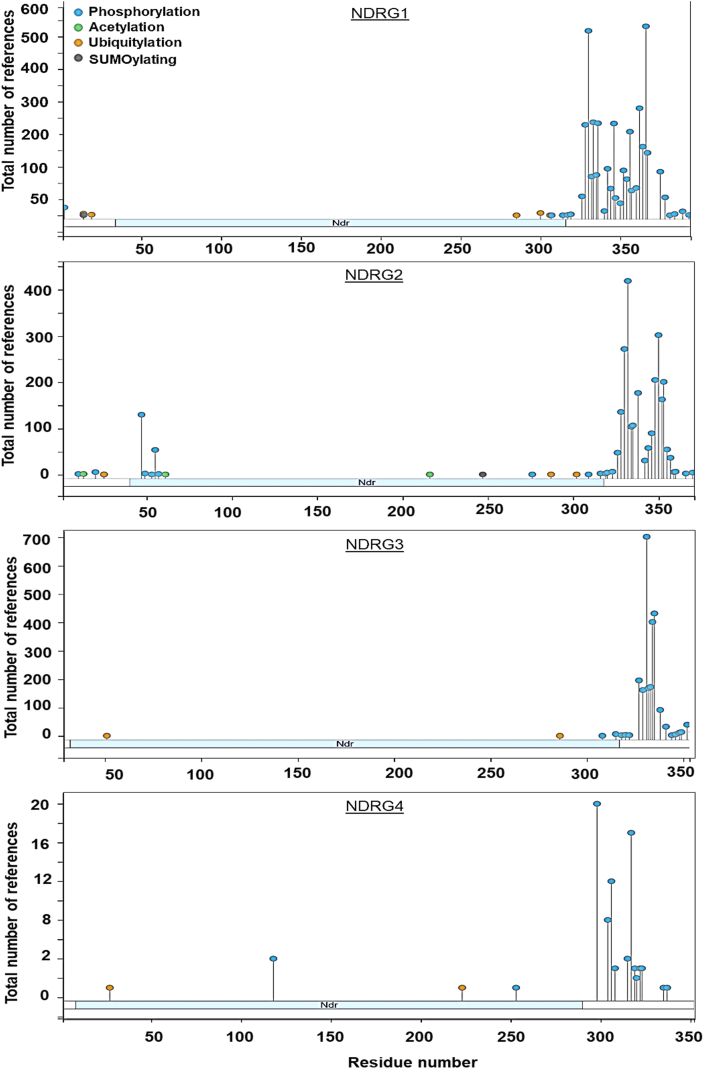
**Phosphoproteomics of NDRG family proteins using PhosphoSitePlus.** Bioinformatics tool, PhosphoSitePlus provides comprehensive data on experimentally verified phosphorylation sites and other post-translational modification from mass spectrometry data and curated literature (93).

### Tissue distribution of NDRG family members

In humans, NDRG1 has been demonstrated to be expressed in most organ systems, including the digestive, immune, reproductive, and urinary systems (94). The highest levels of *NDRG1* mRNA are observed in the kidney, ovary, and especially the prostate (94). Immunocytochemical studies have demonstrated that NDRG1 is mainly expressed in human epithelial cells *e.g.* follicular cells of the thyroid, adrenal medulla, and anterior lobe of the pituitary gland (94). In addition to the brain, human *NDRG2* is also highly expressed in adult skeletal muscle and salivary gland while being moderately expressed in the liver, kidney, heart, adrenal gland, and trachea (30). *NDRG3* is expressed in the brain and testis and moderately expressed in the kidney, prostate, and pituitary gland, while *NDRG4* is expressed in the adrenal and pituitary glands (30).

Based on UniGene cluster analysis of the human genes, *NDRG1, NDRG2*, *NDRG3*, and *NDRG4* are located on chromosome 8q24.22, 14q11.1 to 11.2, 20q12 to 11.23, and 16q21 to 22.1, respectively (95). Northern and dot blot analysis demonstrated that *NDRG2*, *NDRG3*, and *NDRG4* are highly expressed in adult human brain and almost undetectable in eight human cancer cell lines (HL-60, HeLa S3, K-562, MOLT-4, Raji, Daudi, SW480, and A549) (30).

Although the NDRG family is highly expressed in the human adult brain, its members demonstrate different tissue distribution (30, 94). A study conducted by Okuda and associates identified differences in mRNA expression patterns between *NDRG1-3* in the central nervous system (CNS) of mice (17). *NDRG3* was found to be expressed early in the embryological phase on embryonic day 9.5 (E9.5), while *NDRG1* and *NDRG2* expression occurred at approximately E12.5 and E13.5, respectively (17). *NDRG1* expression was mainly observed in the cerebral cortex (17); while the expression of *NDRG2* was relatively low, it was also demonstrated to be widespread in the mouse midbrain, cerebellum, and pons (96). However, *NDRG3* showed a broader expression pattern, including the cerebral cortex and spinal cord of the mouse (17).

The differences in expression patterns of *NDRG1-3* described above in terms of embryonal CNS development suggest possible divergent functional roles (44). Similar differences in *NDRG* family member expression in the CNS were also observed in the frog, *X. tropicalis* (36). In fact, *NDRG1* expression was predominantly identified in the *Xenopus* forebrain, which later develops into the cerebrum, whereas expression of *NDRG2*, *NDRG3*, and *NDRG4* was demonstrated in the developing brain and spinal cord (36). In addition to tissue-dependent expression differences in the four NDRG family members, there were also differences in their subcellular distribution, which are described below.

### Subcellular distribution of NDRG family members

Studies examining the sub-cellular distribution of the NDRG family members remain fragmentary and incomplete. As such, in comparison to data obtained by direct experimental investigation, a well-characterized prediction tool, namely, PSORT II (97), was also was utilized to assess the subcellular distribution of NDRG family members. Investigations into the cellular distribution of NDRG1 have demonstrated that it localized in various cellular compartments, including the cytoplasm, nucleus, plasma membrane, mitochondrion, peri-nuclear region, vesicles, and centrosomes (69). PSORT II predictions suggest a high probability of cytoplasmic localization for NDRG1 (39.1%), NDRG2 (43.5%), NDRG3 (39.1%), and NDRG4 (30.4%). These predictions agree with the current literature indicating that NDRG1 is broadly distributed throughout the cell, with significant cytoplasmic localization (98, 99, 100).

Importantly, PSORT II provides a probability estimate and not actual cellular abundance of localization based on sequence features (97). For instance, it predicts a higher likelihood of mitochondrial localization for NDRG1 (34.8%) and NDRG4 (30.4%) relative to NDRG2 (17.4%) and NDRG3 (13.0%). Considering this, a number of studies have revealed that the NDRG family of proteins could be involved in regulating mitochondrial function (101, 102, 103). PSORT II predicts that the probability of nuclear localization was more prominent for NDRG2 (30.4%) and NDRG3 (39.1%) than NDRG1 (17.4%) and NDRG4 (4.3%).

DNA damage results in the nuclear translocation of NDRG1, which was suggested to implicate a role in DNA repair in a p53-dependent manner (104). Moreover, as highlighted previously, phosphorylated NDRG1 (Ser330) is predominantly localized in the nucleus, while NDRG1 phosphorylated at Thr346 was largely cytoplasmic (69). Comparing the cytoplasmic and nuclear levels of full-length and truncated isoforms of NDRG1, the full-length isoform of NDRG1 was localized to the nucleus at higher levels relative to the truncated isoform in all six cancer cell-types mentioned above (69).

While the subcellular distribution of NDRG2, 3, and 4 has not been extensively characterized, NDRG2 in mouse astrocytes is widely distributed in the cytoplasm and co-localizes with another cytoplasmic protein and astrocytic marker, glial fibrillary acidic protein (GFAP) (105). However, NDRG2 has also been found in the nucleus of glial cells (106). In a mouse model of ischemic stroke, NDRG2 protein expression was markedly increased in the nucleus of astrocytes in ischemic penumbra at 24 h, while in the control, NDRG2 was predominantly localized in the cytoplasm (107). This effect has been hypothesized to be an apoptotic response induced by ischemia–hypoxia injury (107). NDRG3 has been demonstrated to be predominantly localized in the nucleus of most cerebral neuronal cells of mice, while NDRG4 was primarily distributed in the cytoplasm of neurons and Purkinje cells of the cerebellum (105).

### Molecules that interact with NDRG family members

Molecular interactions between proteins are often essential for their biological functions. NDRG1 has been demonstrated to have many molecular partners, as highlighted in Table 2. This aspect is further discussed below implementing a number of pertinent examples.

**Table 2:** Reported potential NDRG1-binding proteins

Protein name	Function	References
Androgen receptor, AR	Steroid hormone receptor	(111, 307)
Heat shock protein 90, HSP90	Molecular chaperone	(11, 307)
Mitogen-inducible gene 6, MIG6	Negative regulator of the EGFR, Tumor suppressor	(128)
E-cadherin	Cell adhesion protein	(231, 307)
β-catenin	Cell adhesion protein and transcription co-activator in the WNT signaling pathway	(123, 307)
c-Myc	Transcription factor	(308)
Protein Kinase C α, PKCα	Serine/threonine-protein kinase	(123)
Low-density lipoprotein receptor-related protein, LRP6	Co-receptor in WNT pathway	(7)
Serum/glucocorticoid-regulated kinase-1, SGK1	Serine/threonine-protein kinase	(82)

#### Heat shock protein 90 (HSP90)

HSP90 is a molecular chaperone responsible for the maturation and activation of many proteins (108). It interacts with misfolded proteins and prevents their aggregation of these *via* an ATP-dependent mechanism (109). There are two isoforms of HSP90 expressed in the cytosol and nucleus (HSP90α and HSP90β), which are essential for HSP90 dimer formation and are responsible for its function (109).

It has been demonstrated by Banz *et al.* that HSP90 and NDRG1 physically associate in hepatocellular cancer cells, resulting in the stabilization of NDRG1 (110). Further, the transcription and phosphorylation of NDRG1 may be regulated by HSP90, as it was demonstrated that the inhibition of HSP90 activity resulted in a transcriptional increase of NDRG1 levels (110). It was also discovered that HSP90 regulates NDRG1 regulating kinases such as glycogen synthase kinase-3β (GSK3β) and SGK1, as HSP90 inhibition decreased GSK3β and SGK1 protein levels, subsequently leading to decreased NDRG1 threonine phosphorylation (110). However, the inhibition of HSP90 activity did not lead to HSP90/NDRG1 complex dissociation, and it did not affect NDRG1 protein stability (110). This later observation suggests a non-canonical interaction between HSP90 and NDRG1. More recently, NDRG1 has been shown to interact with the HSP90 and androgen receptor (AR) complex, which prevents AR activation (111).

#### Glycogen synthase kinase-3β (GSK3β)

GSK3β is a ubiquitously expressed protein kinase, which participates in many cellular functions ranging from signaling transduction to cellular differentiation (112, 113). GSK3β is also demonstrated to regulate tumor progression, invasion, and metastasis and may play an oncogenic or anti-oncogenic role (114, 115, 116, 117). Additionally, GSK3β may also participate in the resistance of cancer cells to chemotherapy and radiotherapy (118, 119). The diverse functions of GSK3β are mediated through its numerous downstream targets (112, 114, 120, 121). As mentioned above, HSP90 regulates NDRG1 transcription and phosphorylation through GSK3β, which indicates that NDRG1 is one of the downstream targets of GSK3β (110).

Furthermore, NDRG1 directly interacts with GSK3β to participate in the regulation of β-catenin degradation in hepatocellular carcinoma cells (26). The association of NDRG1 with GSK3β prevents the interaction between GSK3β and β-catenin, which rescues β-catenin from phosphorylation and subsequent degradation mediated by the destruction complex, enhancing nuclear β-catenin translocation (26). This conclusion is supported by observations that β-catenin protein levels and nuclear accumulation decrease after suppressing NDRG1 expression in hepatocellular carcinoma cells. This effect leads to reduced expression of the downstream transcriptional target of β-catenin, *cyclin D1* (26, 122).

While these latter studies were reported using hepatocellular carcinoma cells, NDRG1 regulatory activity differs in pancreatic cancer cells, prostate cancer cells, and colon cancer cells (11, 123, 124). In these later cell types, GSK3β expression or activation was either not affected by NDRG1 expression in prostate and colon adenocarcinoma cancer cells (124) or downregulated and inactivated by NDRG1 expression in pancreatic cancer cells (123). Under the latter conditions, there was inhibition of β-catenin nuclear translocation and its targeting to the plasma membrane (11, 123, 124). Collectively, these studies highlight the pleiotropic role of NDRG1 in its differential interaction with GSK3β depending on the cell type.

#### Mitogen-inducible gene 6 (MIG6)

Mitogen-inducible gene 6 (MIG6) is encoded by the gene, *ERRFI1*, with its function being a well-characterized epidermal growth factor receptor (EGFR) inhibitor (125) and tumor suppressor (126). In fact, the association of MIG6 with EGFR leads to inhibition of its kinase activity (127), but also to the internalization of EGFR into endosomes and later into lysosomes for degradation (128, 129). Overexpression of NDRG1 in pancreatic cancer cells results in MIG6 stabilization, with MIG6 half-life increasing from 1.6 ± 0.2 h under control conditions to 7.9 ± 0.4 h after NDRG1 overexpression (128). The increased MIG6 levels enhanced EGFR co-localization with the late endosome/lysosomal marker, lysosomal-associated membrane protein 2 (LAMP2).

An increase in EGFR levels after *MIG6* silencing was evident after NDRG1 overexpression, which was consistent with a functional role for MIG6 in NDRG1-mediated downregulation of EGFR (128). Conversely, the silencing of *NDRG1* was employed to further validate the findings and prevent artefactual results associated with NDRG1 overexpression models. Silencing *PTEN*, which plays a role in early to late endosome maturation, decreased MIG6 levels, rescuing EGFR with or without NDRG1 overexpression (128). These results suggest a role for PTEN in regulating MIG6 levels (128). Anti-tumor drugs of the di-2-pyridylketone thiosemicarbazone class that upregulate NDRG1 markedly increased MIG6 and led to its cytosolic co-localization with NDRG1 (128). This was accompanied by decreased total and activated EGFR and its redistribution to late endosomes/lysosomes. As such, NDRG1 induces EGFR downregulation *via* MIG6, leading to late endosomal/lysosomal processing of EGFR (128).

MIG6 has also been demonstrated to inhibit HER2, HER3, and HER4 expression in esophageal cancer (130). The broad inhibitory action of MIG6 was also demonstrated on the activity and expression of other receptor tyrosine kinases such as c-Met (131). In fact, upregulation of MIG6 as a result of NDRG1-inducing agents, such as *di-2-pyridylketone 4,4-dimethyl-3-thiosemicarbazone* (Dp44mT) and di-2-pyridylketone-4-cyclohexyl-4-methyl-3-thiosemicarbazone (DpC), resulted in decreased total c-Met protein and phosphorylation as a result of lysosomal degradation (132). This observation was further validated upon *MIG6* silencing, which abrogated c-Met downregulation in prostate cancer cells (132).

#### Protein kinase Cα (PKCα)

Recent studies discovered a role for NDRG1 in the regulation of the Wingless (WNT)/β-catenin signaling pathway *via* its ability to upregulate protein kinase Cα (PKCα) in pancreatic cancer cell types (123). This regulation was also apparent in multiple other tumor cells, including melanoma, breast cancer, and neuroblastoma cells, indicating the relationship is broadly observed (123). These studies indicated that NDRG1 overexpression in pancreatic cancer cells resulted in a marked decrease in total GSK3β protein levels and its activation (123). In contrast, the destabilizing phosphorylation of β-catenin at Ser33, Ser37, and Thr41 that is classically mediated by GSK3β was significantly increased after NDRG1 overexpression, suggesting a GSK3β-independent mechanism (123). The overexpression of NDRG1 also upregulated PKCα, with *PKCα* silencing inhibiting β-catenin phosphorylation at Ser33, Ser37, and Thr41, which prevented the decrease in β-catenin levels (123).

These latter experiments indicated a role for PKCα in the phosphorylation of β-catenin (123) (Fig. 3*A*). The association of NDRG1 and PKCα was identified upon NDRG1 overexpression, with PKCα stabilization occurring and its half-life increasing from 1.5 h to 11.0 h (123). Further, an association between PKCα, NDRG1, and β-catenin was identified, with the formation of a potential metabolon that could promote β-catenin phosphorylation at Ser33, Ser37, and Thr41, leading to its downstream proteasomal degradation (Fig. 3*A*) (123). As such, the formation of this metabolon that promotes β-catenin degradation and inhibits its nuclear translocation may be important for NDRG1 anti-oncogenic activity.

**Figure 3:**
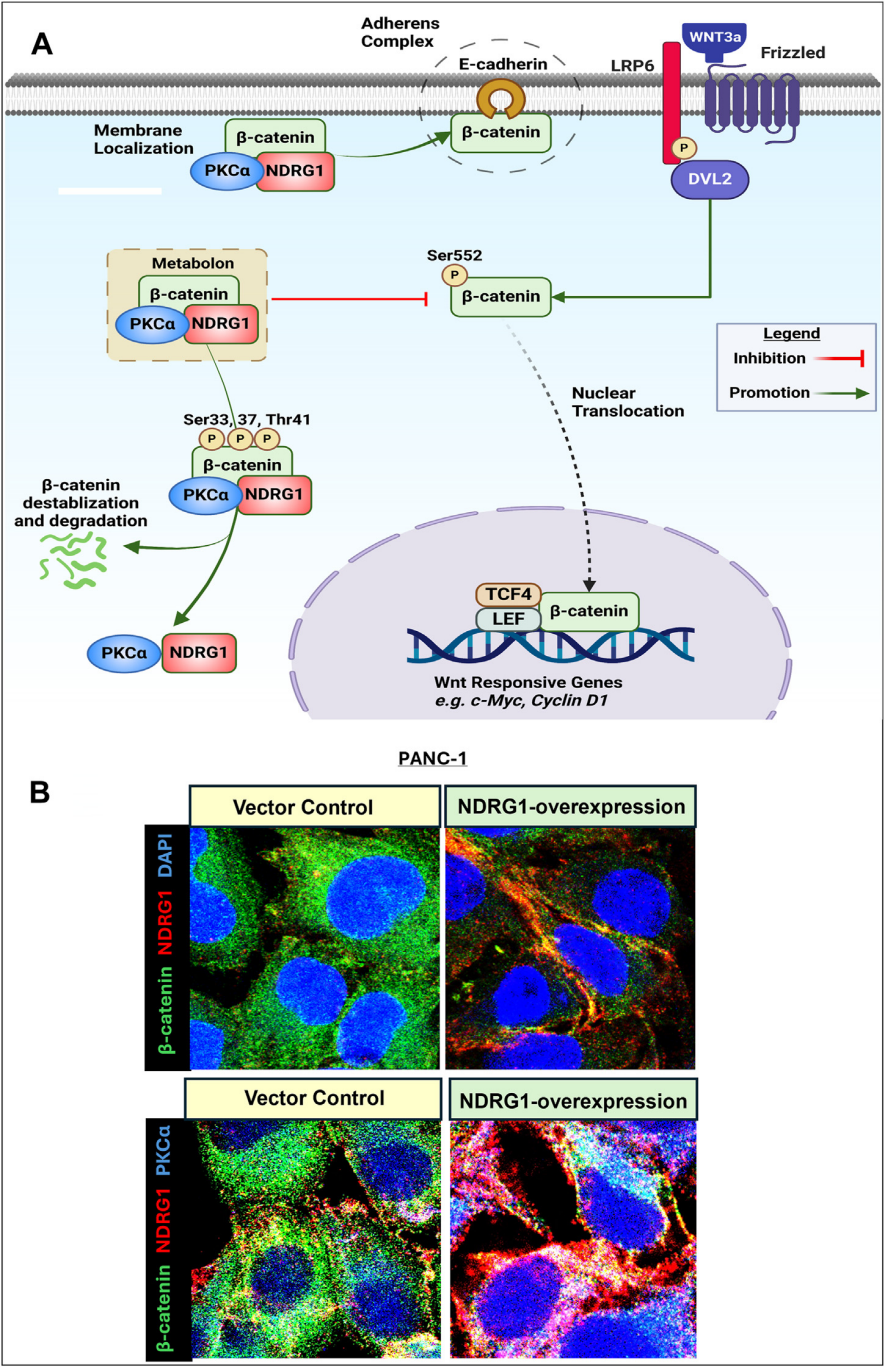
**NDRG1’s novel role in the regulation of β-catenin *via* the formation of a regulatory metabolon between NDRG1, PKCα, and β-catenin in pancreatic cancer cells.***A*, schematic demonstrating the novel WNT/β-catenin regulatory mechanism employed by NDRG1 to decrease nuclear oncogenic β-catenin levels *via* PKCα. This kinase activity of PKCα results in the destabilizing phosphorylation of β-catenin at Ser33, 37, and 41, leading to β-catenin down-regulation (123) *via* proteasomal degradation. This effect prevents the nuclear translocation of β-catenin. Instead, some β-catenin translocates to the plasma membrane after NDRG1 overexpression and is co-localized with both NDRG1 and PKCα. The increased β-catenin levels at the plasma membrane aid adherens complex formation composed of β-catenin and E-cadherin that prevents the epithelial-mesenchymal transition (209, 244). *B*, confocal microscopy analysis of PANC-1 vector control and NDRG1-overexpressing pancreatic cancer cells demonstrating co-localization (*yellow*) between NDRG1 (*red*) and β-catenin (*green*). This observation suggests association between NDRG1 and β-catenin mostly localized at the plasma membrane. Similarly, immunostaining of NDRG1 (*red*), PKCα (*blue*), and β-catenin (*green*), demonstrates triple-colocalization (*white*). The scale bar is 3 μm. This finding, together with co-immunoprecipitation evidence, suggests a ternary complex of three proteins, potentially indicating the formation of a metabolon localized predominantly in the cytoplasm and plasma membrane. *A*, was created in BioRender (Azad, M. (2025) https://BioRender.com/d18u703). *B*, is from the authors publication (123) under the terms of the Creative Commons CC-BY license.

The effect of genetically induced NDRG1 expression was also mimicked by the NDRG1-inducing thiosemicarbazones, Dp44mT and DpC, highlighting their therapeutic potential (123). An intriguing aspect of the effect of NDRG1 on PKCα and β-catenin expression and cellular distribution was the demonstration of NDRG1, PKCα, and β-catenin at the plasma membrane using confocal microscopy (123). This interaction between NDRG1 on PKCα and β-catenin was further confirmed by co-immunoprecipitation (123) (Fig. 3*B*). This was notable, as β-catenin is a critical structural component of the adherens complex at the plasma membrane that promotes cellular adhesion and inhibits migration and metastasis (11). Furthermore, NDRG1-induced localization of β-catenin at the plasma membrane has also been reported by our lab in other cell types (11, 124). These findings suggest that PKCα and NDRG1 can be localized at the plasma membrane in association with β-catenin (123).

### Transcriptional regulation of NDRG family members

#### HIF-1-mediated transcriptional regulation

Hypoxia plays a significant role in the regulation of NDRG family members (133). It has been well-established that NDRG1 is regulated *via* the transcription factor, hypoxia-inducible factor 1 (HIF-1) (134, 135) (Fig. 4). It is well-documented that HIF-1 plays a pivotal role in the cellular response to low oxygen levels or hypoxia (136). Under normoxia, the HIF-1α subunit of HIF-1 is continually synthesized, ubiquitinated, and degraded *via* the proteasomal pathway (137, 138, 139). This process of degradation is due to the oxygen-dependent hydroxylation of HIF-1α by iron-dependent enzymes, known as prolyl hydroxylases (PHDs), which function to hydroxylate prolyl residues in HIF proteins (140, 141, 142). These residues are then recognized by the von Hippel-Lindau protein (pVHL), a protein within the E3 ubiquitin ligase complex that marks HIF-1α for ubiquitination (140, 141, 142) (Fig. 4). However, under hypoxic conditions, the hydroxylation of these residues is inhibited due to reduced oxygen availability, which is critical for PHD function (136, 143). Hypoxia or hypoxia-mimicking compounds such as iron chelators, nickel, or cobalt ions increase NDRG1-4 expression at the transcriptional level *via* HIF1α-dependent and -independent mechanisms (42, 134, 144, 145, 146, 147). Of interest, nitric oxide (NO) has also been demonstrated to upregulate NDRG1 expression in triple-negative breast cancer cells (HCC 1806) and inhibit cellular migration (148). The mechanism behind this effect was due to the ability of NO to bind cellular iron pools, leading to its release as dinitrosyl dithiol iron complexes that had been demonstrated using other cell types (149, 150, 151). These studies in breast cancer cells indicated for the first time a link between NO, chelatable iron pools, cellular signaling, and NDRG1 expression (148).

**Figure 4:**
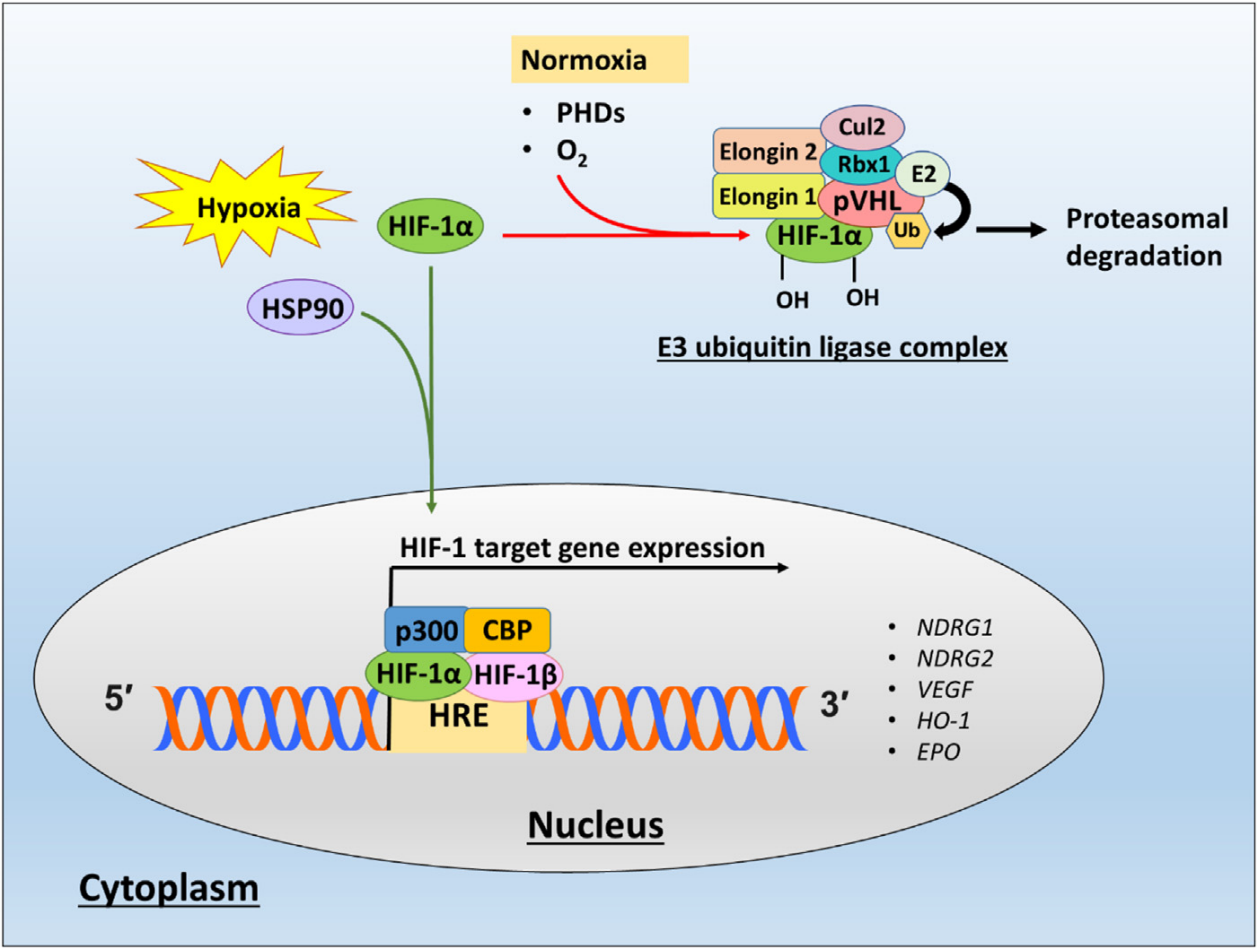
**The hypoxia-inducible factor-1α (HIF-1α) mediated transcriptional regulation of *NDRG1* expression.** Under normoxia, the transcription factors, HIF-1α and HIF-2α, are ubiquitinated and degraded *via* the proteasomal pathway. Prolyl hydroxylase (PHD) enzymes require sufficient oxygen levels to hydroxylate prolyl residues within HIF-1α and HIF-2α, which can then be recognized by the von Hippel-Lindau protein (pVHL), a E3 ubiquitin ligase complex that ubiquitinates HIFs. Under conditions of hypoxia, the low oxygen levels suppress PHD enzyme activity leading to increased protein levels of the HIFs. HSP90 stabilizes HIF-1α and prevents pVHL-dependent proteasomal degradation. The binding of HSP90 also changes the conformation of HIF-1α so that it can recruit the cofactor p300. The interaction of HIF-1α with p300 and CBP promotes the activation of hypoxia-response elements (HREs) within the promoter and enhancer regions of target genes such as *NDRG1*.

The molecular chaperone, HSP90, stabilizes HIF-1α, prevents pVHL-dependent proteasomal degradation, and changes the conformation of HIF-1α to recruit the cofactor, p300 (152, 153, 154) (Fig. 4). This stabilization by HSP90 promotes translocation of HIF-1α to the nucleus, where it dimerizes with HIF-1β to activate hypoxia-response elements (HREs; 5′-RCTCG-3′) that are involved in HIF-binding within the promoter and enhancer regions of target genes such as *NDRG1* (134, 155, 156). In fact, *NDRG1* has three active HREs within its enhancer region (134), with one located within the promoter region and two more in the 3′ untranslated region (134, 135). Studies suggest the region between −1202 to −450 of the *NDRG1* promoter is the most critical for HIF-1α binding, thereby enhancing the transcriptional upregulation of *NDRG1* (135, 145).

Studies by Wang *et al.* identified *NDRG2* as a HIF-1 target gene in A549 lung cancer cells (42). In this latter study, it was revealed that, similar to NDRG1, *NDRG2* mRNA and protein levels were increased in several tumor cell lines exposed to hypoxia (42). Three HREs have been identified within the *NDRG2* promoter, with HIF1 directly interacting with the HREs (42). While NDRG1 and NDRG2 exhibit similar HIF1-mediated transcriptional regulation *via* the HIF1-HRE mechanism (134, 135), NDRG3 and NDRG4 are regulated indirectly by hypoxia (147). In fact, as described previously, NDRG3 has been suggested to be regulated post-translationally in hypoxic cancer cells *via* lactate binding (147). Oxygen levels negatively regulate NDRG3 protein levels through the PHD2/VHL system, and excess lactate generated during prolonged hypoxia impedes the proteasomal degradation of NDRG3 *via* direct association with this protein (147).

A mechanism involving the regulation of NDRG4 by HIF-1 has yet to be elucidated. However, in studies subjecting rat cardiomyocytes to hypoxia and re-oxygenation, NDRG4 expression was shown to increase (34). In this latter investigation, *NDRG4* was transcriptionally upregulated by TNF-α/NF-κB signaling, which further indicates that NDRG4 may be indirectly regulated by hypoxia.

#### Myc-mediated transcriptional regulation

Myc-dependent regulation of the NDRG family has been extensively explored over the past several decades (157). These studies screened Myc target genes in *MYCN*-mutated mouse embryos and, after cDNA subtraction, identified in mutant embryos a 20-fold amplification of the *NDRG1* gene (157). Studies identified that co-expression of N-Myc and its key protein partner, MAX, led to the suppression of NDRG1 expression, with later reports highlighting the negative regulatory effect of other Myc family members on NDRG1 activity, namely c-Myc (157).

While the regulation of NDRG3 and NDRG4 by Myc has yet to be elucidated, there is evidence to suggest that, like NDRG1, NDRG2 is similarly regulated by the Myc protein family (158). A 2006 study demonstrated that NDRG2 is downregulated by c-Myc *via* transcriptional repression, with the ectopic expression of c-Myc resulting in the suppression of NDRG2 mRNA and protein levels (158). It was also further demonstrated that Myc interacts with the core promoter region of *NDRG2 in vitro* and *in vivo*, and that this c-Myc-mediated repression of *NDRG2* transcription required MIZ-1 (158). Considering these data, it has been suggested that NDRG2 may play regulate cellular differentiation (158).

#### p53-mediated transcriptional regulation

The tumor suppressor, p53, is commonly referred to as the ‘guardian of the genome’ due to its ability to prevent the development of cancer in response to DNA damage (159, 160). This ability is mediated *via* it inducing cell cycle arrest, apoptosis, and/or senescence (161). p53 regulates NDRG1 levels *via* acting as a transcription factor by binding to *NDRG1* promotor region, promoting *NDRG1* transcription (162). It has been demonstrated in some cell types that NDRG1 expression is induced by DNA damage in a p53-dependent manner (134, 161, 162, 163).

Studies using RNA interference and inducible gene expression have proposed that NDRG1 was required for p53-mediated caspase activation and apoptosis in response to DNA damage (163). However, other investigations have not demonstrated any effect of the DNA-damaging agents, actinomycin D, mitomycin C, and cisplatin, on cellular NDRG1 levels in MCF-7 cells that possess wild-type p53 (134). This lack of effect of these agents on NDRG1 expression was despite their ability to upregulate growth arrest and DNA damage 45 (GADD45) and wild-type p53 activating fragment 1 (*WAF1*) mRNA (134) that are known p53-responsive DNA damage genes (164, 165).

Although there is limited evidence for the regulation of NDRG1 by p53 under some conditions, the role of p53 in regulating other NDRG family members is yet to be fully elucidated. An investigation in 2013 explored the role of NDRG2 as a novel p53-associated regulator of apoptosis in IL-6-differentiated C6 glioma cells exposed to oxygen- and glucose-deprivation (166). This latter study demonstrated that NDRG2 overexpression inhibited cellular proliferation and increased Bax/Bcl-2 ratios, with *NDRG2* silencing having the opposite effect (166). The silencing of *p53* caused a significant decrease in oxygen and glucose-deprivation-induced apoptosis and NDRG2 levels, while p53 overexpression did not affect NDRG2 expression (166). This latter study indicated a potential role of p53 in the regulation of NDRG2, and a possible role in p53-mediated differentiation and proliferation. However, a detailed understanding of the role of p53 in regulating NDRG2 expression remains unclear.

### NDRGs, autophagy, and calcium signaling

#### Role of NDRG family members in calcium signaling and lysosomal biogenesis

Calcium is an essential second messenger/signaling molecule that regulates a wide variety of biological functions (167). Calcium signaling plays a role in many significant cellular functions, such as apoptosis, proliferation, differentiation, and gene transcription (167). The deregulation of calcium-dependent pathways is also implicated in several diseases (167). NDRG1 was earlier known as Cap43, as it is regulated and activated by Ca^2+^ (168, 169). An increase in free intracellular calcium was suggested to increase NDRG1 expression as part of the HIF1α-independent hypoxic response (15, 169, 170). Cellular nickel levels have also been demonstrated to promote the release of stored intracellular calcium, which in turn enhances NDRG1 expression (169).

There have been limited studies investigating the link between NDRG1 and increased calcium levels or calcium trafficking within cells (168). One such investigation examined the link between NDRG1 expression and the endoplasmic reticulum (ER) stress response (168). It was demonstrated that the upregulation of NDRG1 in pancreatic cancer cells modulated the ER stress response by increasing the expression of ER calcium-binding chaperones, such as calreticulin and calnexin (168). The same investigation also examined cytosolic calcium levels after NDRG1 overexpression or after pharmacological induction of NDRG1 using Dp44mT (168). These results demonstrated a significant increase in cytosolic calcium levels, particularly after the combination of genetic NDRG1 overexpression and pharmacological induction of NDRG1 by Dp44mT (168). It was postulated that the anti-proliferative activity observed using Dp44mT, in part, could be a result of an increase in cytosolic calcium levels, which increased sensitivity to apoptosis through the activation of CaMKII signaling (168).

There are also limited studies on other NDRG family members and their link with cellular Ca^2+^ levels. However, a study by Chen *et al.* demonstrated that NDRG2 expression in MC3T3-E1 pre-osteoblast cells promoted osteoblastic differentiation and calcification stimulated by Bone morphogenetic protein 2 (BMP2) *via* the activation of the JAK/STAT3 signaling pathway (171). It was highlighted that NDRG2 overexpression in MC3T3-E1 cells promoted intracellular calcium salt deposition, with this effect being reversed upon suppressing NDRG2 expression (171).

#### NDRGs and their link to autophagy and lysosomal biogenesis

Autophagy is a cellular process that facilitates the recycling of cytoplasmic components, including misfolded or damaged proteins, in response to various stresses such as nutrient deprivation, metabolic stress, and tumorigenesis (172). Autophagy is a dynamic process consisting of the initiation stage, involving the formation of the phagophore and autophagosomes, followed by the degradation stage, where the autophagosomes fuse with lysosomes (173, 174, 175). Autophagy plays a complex role in cancer cells as it has been demonstrated to act: (1) as a regulator of key oncogenes and tumor suppressor genes to facilitate tumor development; (2) have cytoprotective roles (176); and (3) induce cancer cell death (177).

The autophagic pathway has been implicated to play a key role in tumor cell growth and metastasis (178, 179). Considering that autophagy is regulated by stress generated both extracellularly and intracellularly, and that NDRG1 is also a stress response protein (31, 66, 168), it was of interest to investigate the relationship between NDRG1 and autophagy (180). It has been described by our laboratory that NDRG1 expression is involved in the negative regulation of autophagy at the initiation level in pancreatic cancer cells (181). As postulated by Wang *et al.*, NDRG1 could play a key role in the formation and the proteolytic function of lysosomes, which are important as part of the autophagic “machinery” (182). Furthermore, *NDRG1* knockdown reduced the fusion ability of the autophagosomes with the lysosomes (183), which in turn resulted in reduced autophagosome turnover.

NDRG1 may be associated with lysosomes, due to colocalization between NDRG1 and the lysosomal marker, LAMP1 (183). Further, it was highlighted that *NDRG1* silencing resulted in defective lysosomal activity, which was characterized by decreased proteolytic activity and increased lysosomal pH from 4.3 to 5.4 (183). While the latter study is interesting, it is unclear if the change observed is physiologically significant, as a relatively broad pH range has been ascribed to the lysosomal microenvironment, *i.e.*, pH 5 to 5.5 (184).

##### The role of NDRG1 in the regulation of autophagy: Effects on autophagic initiation and degradation

As alluded to above, studies have demonstrated that NDRG1 inhibits autophagic initiation by a mechanism involving the suppression of the protein kinase RNA-like endoplasmic reticulum kinase (PERK)/eIF2α pathway (180). In pancreatic cancer cells, treatment with the cytotoxic chelator, Dp44mT, increased ER stress leading to the phosphorylation of PERK and eIF2α as well as microtubule-associated protein 1 light chain 3 (LC3) II levels that are a marker of autophagosomes and autophagic initiation (180). Interestingly, this effect of Dp44mT could be suppressed upon genetic *NDRG1* overexpression (180). Hence, while Dp44mT upregulates NDRG1 expression *via* cellular iron depletion (134), it also induces the ER stress response and autophagic initiation (180). This stress response was due to the ability of Dp44mT not only to bind cellular iron, but also result in oxidative stress due to the formation of redox active iron and copper complexes (185, 186) (Fig. 5).

**Figure 5:**
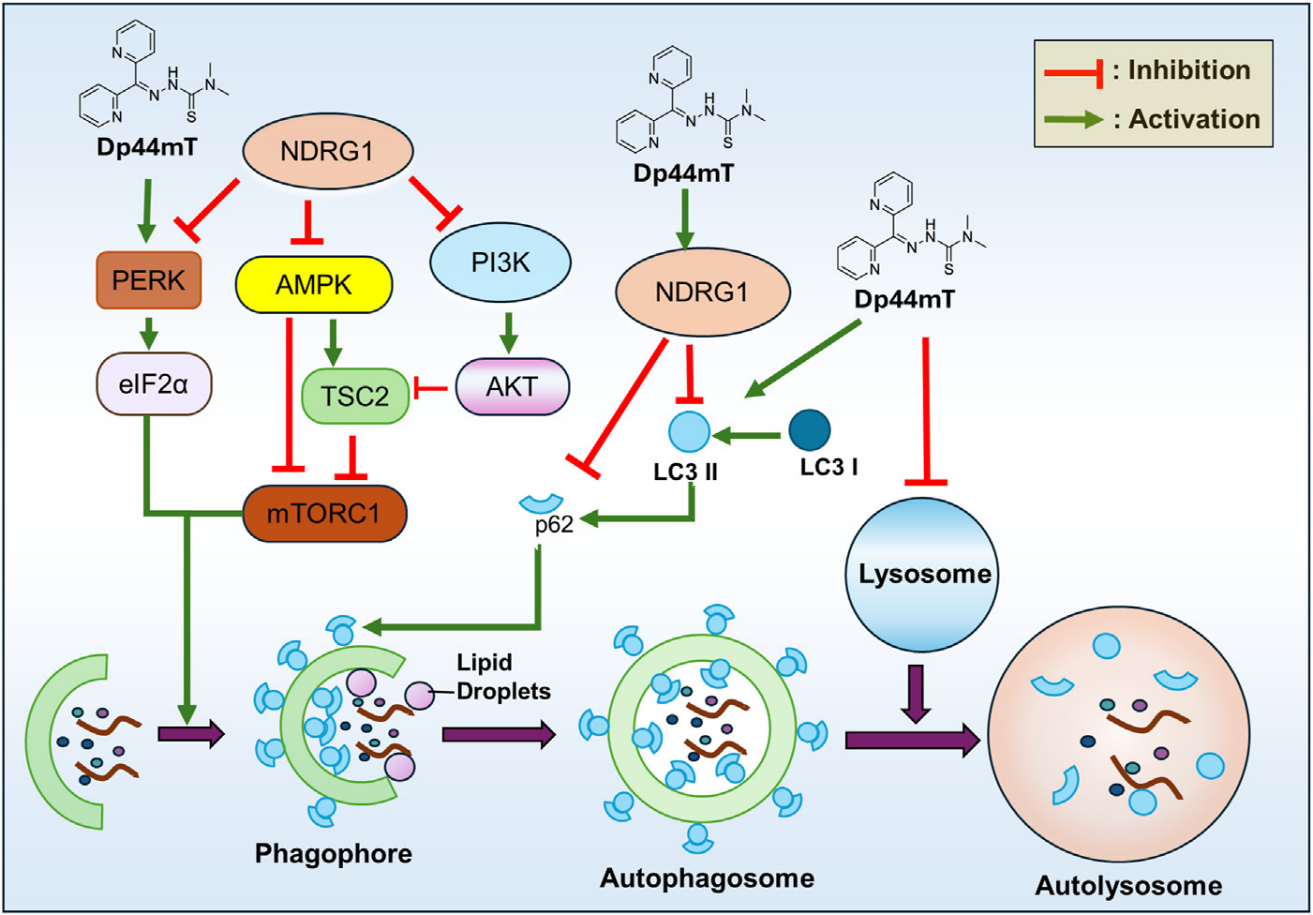
**The role of NDRG1 on the regulation of autophagy.** NDRG1 suppresses autophagy at both the initiation and degradation stages. It suppresses autophagic initiation by suppression of the PERK/eIF2 and PI3K/AKT pathways, as well as *via* AMPK (83, 181, 193, 300). The pharmacological NDRG1-inducing agent, Dp44mT, up-regulates autophagic initiation leading to increased LC3II levels *via* the PERK/eIF2α axis (180). NDRG1 expression also up-regulates p62 levels, suggesting its suppressive role in autophagic degradation (181). However, Dp44mT promotes lysosomal membrane permeabilization due to the generation of reactive oxygen species after the formation of redox-active iron and copper complexes (185, 186). The effect of Dp44mT on inducing initiation while damaging lysosomes results in dysfunctional autophagy. Similarly, NDRG1 may suppress lipophagy *via* downregulating LC3II while increasing p62 expression (191).

Furthermore, the latter investigation also demonstrated the inhibition of pro-survival autophagy by NDRG1 led to an increase in pro-apoptotic markers such as cleaved caspase 3 and caspase 4, while anti-apoptotic Bcl-2 expression was suppressed (180). Hence, it was suggested the ability of NDRG1 to reduce autophagic flux results in decreased recycling of essential nutrients such as iron that could play a role in the ability of this metastasis suppressor to trigger apoptosis and inhibit metastasis. A subsequent study by Wang *et al.* using osteosarcoma cells demonstrated that the inhibition of NDRG1 expression increased LC3II-positive autophagosomes *via* the suppression of autophagosome fusion with lysosomes (183).

Considering the role of NDRG1 in the degradation stage of autophagy, studies have investigated the effect of NDRG1 on p62 expression, which is encoded by *sequestosome 1* (*SQSTM1*) in humans (181), and is also known as the ubiquitin-binding protein p62. The role of p62 is as a cargo receptor that delivers misfolded or damaged proteins for autophagic degradation (187) (Fig. 5). After the delivery of the protein cargo, p62 remains within autophagosomes and is degraded, leading to an inverse relationship between autophagic activity and p62 levels (187). Thus, increased p62 protein levels relate to a decrease in autophagosome-mediated degradation and is a well-known marker of autophagic degradation.

An investigation by Sahni *et al.* demonstrated a significant upregulation of p62 in NDRG1 overexpressing cells, suggesting suppression of the later stages of autophagy by NDRG1, *i.e.*, autolysosome-mediated degradation (181). In fact, these later studies demonstrated that genetically expressed NDRG1 suppressed basal and hypoxia-induced autophagy at both the initiation and degradation stages and sensitized pancreatic cancer cells to lysosomal membrane permeabilization induced by Dp44mT and its redox-active copper complex. These results were in good agreement with previous studies indicating that NDRG1 expression promotes the anti-proliferative and anti-migratory activity of Dp44mT against the same pancreatic cancer cell type (168). This led to the conclusion that NDRG1 promotes a major cytotoxic mechanism of Dp44mT, namely lysosomal membrane permeabilization (188).

Interestingly, NDRG1 has also been shown to play an important role in suppressing lipophagy, a specialized type of autophagy involving the lipolysis of lipid droplets to free fatty acids *via* the fusion of lipid droplets with lysosomes (189, 190, 191). A study by Wang and associates demonstrated that NDRG1-depletion resulted in increased autophagy in MARC-145 monkey kidney cells, as evident by increased LC3 II and decreased p62 levels (191). This work also showed that the knockdown of NDRG1 expression resulted in increased cellular free fatty acid levels, as result of increased lipophagic activity (191). Overall, these later investigations suggest NDRG1 suppresses lipophagy and are consistent with previous studies examining the general inhibitory effect of NDRG1 expression on autophagy (164). Similarly as observed with NDRG1 (181, 183), NDRG2 has been associated with the inhibition of autophagy (192), as decreased NDRG2 levels resulted in increased autophagic activity (192).

##### NDRG1 and its link to the AMPK pathway

As discussed above, NDRG1 suppresses the initiation stage of autophagy, which can be induced by 5′-AMP-activated protein kinase (AMPK) pathway that acts as a metabolic energy sensor to induce catabolism (181, 193). Autophagy can be induced under nutrient restriction (193), with this depletion also resulting in AMPK activation, which then deactivates the mammalian target of rapamycin complex 1 (mTORC1) (194). However, NDRG1 is also known to inhibit the PI3K/AKT pathway (83), a key pathway known for its role in the activation of mTORC1 (195) and the subsequent suppression of autophagy (196). Despite the complexity in the role of NDRG1 on the regulatory pathways of mTORC1 and autophagy, it has been demonstrated that the overexpression of NDRG1 has been associated with the marked inhibition of AMPK activation (181), and this is in good agreement with the inhibition of autophagy under this condition. This inhibition of AMPK favors anabolic mechanisms rather than catabolic processes such as autophagy, and in fact, NDRG1 expression is associated with a cellular anabolic shift toward glycogen synthesis (197) and *de novo* lipogenesis (38).

This metabolic alteration toward anabolism after NDRG1 expression in pancreatic cancer cells was also confirmed by studies examining key enzymes involved in fatty acid and cholesterol synthesis (181). In fact, the rate-limiting enzymes in fatty acid synthesis and cholesterol synthesis, namely, acetyl CoA carboxylase 1 and 3-hydroxymethylglutaryl CoA reductase, respectively, which are key downstream targets of AMPK, were activated in NDRG1 overexpressing cells, favoring fatty acid and cholesterol synthesis. For these enzymes, the inactivating phosphorylation mediated by activated AMPK were decreased, which was in agreement with the reduced AMPK activation after NDRG1 expression (181). However, total cellular cholesterol levels were shown to decrease upon NDRG1 overexpression, possibly through mechanisms independent of AMPK and autophagy (181).

The role of NDRG1 in lipid metabolism is supported by a variety of evidence. This includes the fact that NDRG1 mutations lead to defective myelin formation by Schwann cells (198), and that *NDRG1* silencing in epithelial cells results in decreased cellular uptake of low-density lipoprotein (LDL) due to decreased LDL receptor (LDLR) expression localized at the cell membrane (199). As described above, these findings also agreed with the concept that NDRG1 expression inhibited lipophagy (*i.e.*, lipid droplet catabolism) in other cell-types (191). Similarly to what is observed after NDRG1 expression (181), NDRG2 has also been demonstrated to inhibit glucose deprivation-induced AMPK phosphorylation and activation (200).

## Different roles of the NDRG family in cancer and other diseases

### Roles of the NDRG family in various cancer types

NDRG1 has been widely recognized as a metastasis suppressor that plays a role in various biological processes, including cellular proliferation and differentiation (201, 202). It has been extensively studied, with tumor context-dependent roles being observed in multiple cancer types that are described below and in Table 2 (21, 32, 201).

The role of NDRG1 in breast cancer phenotype remains complex, as reports suggest both pro- and anti-oncogenic roles (203). NDRG1 has been demonstrated to generally act as a metastasis suppressor in hormone receptor-positive BC cells (6). In a clinical study, it was elucidated that NDRG1 is significantly downregulated in patients with lymph node or bone metastasis compared with those with localized breast cancer (6). Subsequently, resulting in the significant inverse correlation of NDRG1 with patient disease-free survival rate, and this was demonstrated as an independent prognostic factor (6). Furthermore, *in vitro* examination of the overexpression of NDRG1 in triple-negative breast cancer cell-line, MDA-MB-463 demonstrated suppressed invasiveness (6). In a study of SCID mice injected with a human bone metastatic breast cancer cell line, MDA-MB231-BoM, treatment with NDRG1-inducing agent Dp44mT prevented metastasis compared to the vehicle control (7). Conversely, the knockdown of *NDRG1* expression in these tumor cells largely prevented the ability of Dp44mT to prevent metastasis (7). Immunohistochemical examination of the tissue from 29 patients with prostate cancer and 33 patients with breast cancer, demonstrated in 7 there was high NDRG1 levels in normal prostate and breast epithelium, whereas a significant down-regulation was observed in high-grade cancers (7). Of note, β-catenin was predominantly localized in membrane/cytoplasm in normal tissues. However, in high grade tumors, membrane-bound β-catenin levels decreased, resulting and increased cytoplasmic and nuclear β-catenin levels (7).

Molecular studies examining triple-negative breast cancer cells have demonstrated that NDRG1 expression can promote the ubiquitination and degradation of receptor tyrosine kinase, HER3, *via* the interaction with the ubiquitin ligases, neural precursor cell expressed developmentally downregulated protein (NEDD4) (204). In contrast, the non-receptor tyrosine kinase, PYK2, that interacts with NEDD4 and HER3, interfered with NEDD4-HER3 binding, with PYK2-NDRG1-NEDD4 forming a circuit that could regulate HER3 degradation and expression (204).

In contrast, there is evidence of NDRG1 being highly expressed in brain metastatic breast cancer and n breast cancer, the latter being a rare tumor that occurs after tumor cells embolize and block lymphatic vessels (19, 205). Animal studies using SCID/Beige, injected with low-NDRG1 expressing or high-NDRG1 MDA-IBC3 inflammatory breast cancer cells, demonstrated increased brain metastasis, greater tumor burden, and reduced survival in mice injected with high-NDRG1 expressing cells compared to their low-NDRG1 expressing counterparts (19). *In vivo* studies using *NDRG1*-knockdown breast cancer cells (SUM149 and MDA-IBC3) demonstrated significantly reduced tumor volumes and inhibited brain metastasis *versus* control. Using gene set enrichment and immunostaining analyses, the authors revealed increased PI3K-AKT-mTOR signaling with increasing phospho-NDRG1 (Tyr346) levels (19). While this association in inflammatory breast carcinoma is intriguing, this study would have benefited from examining the relationship between other critical regulatory phosphorylation sites of NDRG1, such as Ser330 (69, 80).

Immunohistochemical analysis of patient breast cancer tumors (*n* = 216) to assess NDRG1 expression profile revealed that high-NDRG1 expression was associated with cancer aggressiveness, shorter overall cancer survival, and breast cancer-specific survival (19). In these studies, *NDRG1* mRNA expression was higher in tumor tissues compared to normal tissue and also in higher ER-negative tumors relative to ER-positive tumors. Utilizing multiple publicly available datasets (TCGA and CBioPortal), higher *NDRG1* expression was associated with more aggressive HER2+ and basal-like molecular subtypes than in luminal subtypes. *NDRG1* expression was also observed to be higher in triple-negative breast cancer *versus* non-triple-negative counterparts (19).

Additional investigation indicated that *NDRG1* on chromosome 8q24 was markedly amplified in metastatic and basal-like breast tumors (19). NDRG1 was amplified in 33% of metastatic tumors in the Metastatic Breast Cancer dataset relative to 17% of primary breast tumors from the TCGA invasive breast cancer dataset. A strong trend was demonstrated where NDRG1 was linked to tumor aggressiveness features being most amplified in the basal-like subtype (36%), followed by the HER2+ (25%) and luminal A (2.8%) subtypes (19). Increased *NDRG1* copy number was also correlated with significantly elevated mRNA and protein levels *versus* non-amplified tumors. Overall, these studies demonstrated NDRG1 was positively correlated with poor clinical outcomes and cancer traits associated with aggressiveness (19).

A large meta-analysis examined the relationship between NDRG1 expression and recurrence-free survival in 23 publicly available breast cancer mRNA expression datasets, assessing 3554 patients with breast cancer (38). This analysis revealed that patients with high *NDRG1* mRNA levels exhibited nearly a two-fold greater risk of recurring disease 5 years after diagnosis (38). Further examination of an independent cohort of 295 patients with breast cancer, where metastasis was recorded, evaluated the relationship between metastasis and *NDRG1* mRNA expression. This latter analysis revealed that high NDRG1 is a more consistent marker of a negative outcome than the *c-MYC* oncogene (38). Further analysis using a pairwise mRNA expression-level correlation between *NDRG1* and genes analyzed in the Cancer Genome Atlas (TCGA) data set demonstrated that the estrogen receptor was near the top of the list of negatively correlated genes (38). In contrast, there is a positive correlation between NDRG1 and genes related to angiogenesis, glycolysis, hypoxia, and basal cell lineage in breast cancer (38). This illustrates that high NDRG1 may be more common in estrogen receptor-negative breast cancers (38). These studies concur with research indicating that NDRG1 was part of an angiogenesis-related gene signature correlated with breast cancer metastasis (206). Thus, NDRG1 may be associated with breast cancers with marked glycolytic and angiogenic gene expression at the mRNA level.

Similarly to the studies described above, high NDRG1 levels have been detected in estrogen receptor-negative primary breast cancer and are often associated with lymph node metastasis and advanced clinical stage (207). Kaplan-Meier analysis suggested that patients with breast cancer having positive NDRG1 expression had a markedly worse prognosis than those with NDRG1 negatively expressed (207). A recent investigation has also confirmed that NDRG1 hyperexpression is pro-oncogenic in aggressive ER-negative breast cancer, with higher nuclear NDRG1 localization being detected in brain metastatic tumors relative to primary breast cancer (20).

In patients with prostate cancer, it has been demonstrated that *NDRG1* mRNA and protein expression levels are reduced in patients with prostate cancer, indicating a poor prognosis and higher tumor grade (4). A study conducted involving 148 patients has reported that reduced NDRG1 expression at the cell membrane indicated significantly decreased patient survival outcomes (208), which may be due to a correlation with decreased E-cadherin expression that plays a key role in preventing the EMT (208, 209). NDRG1 has also been demonstrated to inhibit androgen signaling in prostate cancer cells to suppress cellular proliferation and migration (111). Similarly, in pancreatic cancer, NDRG1 was demonstrated as a metastasis suppressor *via* attenuating WNT, transforming growth factor β (TGF-β) and NF-κB signaling (13, 210). Low NDRG1 expression is associated with poor prognosis in patients with pancreatic cancer, and NDRG1 overexpression has been demonstrated to inhibit pancreatic tumor growth (211).

Examining melanoma, immunohistochemical staining studies have revealed that NDRG1 was expressed in the cytoplasm, but not in the nucleus, and its expression was upregulated in the primary melanoma cell line (WM-115) after 1, 4, and 8 h of hypoxia (212). However, *NDRG1* mRNA levels were downregulated in a metastatic melanoma cell line (WM-266-4) compared to primary melanoma cell lines (WM-115) under normoxic conditions (212). The highest NDRG1 staining was detected in intradermal nevi (benign mole), which suggests the anti-metastatic roles of NDRG1 in melanoma.

Another study demonstrated that NDRG2 expression is significantly reduced in HT1080 human fibrosarcoma and B16F10 murine melanoma cell lines, which are known for their enhanced metastatic potential (213). An *in vivo* study has demonstrated that B16F10-NDRG2 cells injected subcutaneously showed slower tumor growth compared to B16F10-mock cells (213). B16F10-NDRG2 cells exposed to hypoxic conditions during *in vivo* tumor growth are more prone to apoptosis than control cells, resulting in slower growth and reduced metastasis (213). This finding aligns with previous observations that NDRG2 expression significantly increases after exposure to hypoxic conditions and similar stresses and is closely linked to hypoxia-induced apoptosis (42). Kim and colleagues demonstrated that NDRG2 expression significantly suppressed tumor invasion by inhibiting MMP activities, an angiogenic switch during carcinogenesis, which is regulated through the NF-κB signaling pathway in melanoma (213).

In fact, NDRG2 has been observed to be downregulated in several cancer types (214, 215). NDRG2 overexpression in breast cancer (BC) significantly inhibited tumor cell growth by attenuating Janus tyrosine kinases (JAK2) and STAT3 pathways (216). Similarly, overexpression of NDRG2 in NB has been demonstrated to inhibit cell proliferation *via* suppression of a proliferation-related protein, cysteine-rich protein 61 (CYR61) (217). In pancreatic cancer, it has been demonstrated that inactivation or reduced expression of NDRG2 is associated with poor prognosis (218).

Compared with other NDRG members, NDRG3 is less studied, with limited evidence showing that it can act as a tumor promoter in patients with gastric cancer and is associated with histologic grade and poor patient survival (219). The expression of NDRG3 has also been demonstrated to be related to poor patient outcomes in invasive breast cancer (220). A study has detected that NDRG3 was upregulated in patient tissues with gastric cancer and contributed to 5-fluorouracil drug resistance in gastric cancer cell lines (221). In chronic myelogenous leukemia, NDRG3 has been demonstrated to associate with imatinib resistance *via* promoting nuclear accumulation of β-catenin (222). However, further studies are needed to elucidate the underlying mechanisms involved in NDRG3 activity and its functional roles in various cancer types.

Limited studies on NDRG4 indicate that it may function as a tumor suppressor in multiple cancer types, including breast cancer (223), esophageal adenocarcinoma (224), colorectal cancer (225), and pancreatic cancer (103). It has been demonstrated that NDRG4 plays a role as a tumor suppressor *via* inhibiting the PI3K/AKT signaling in colorectal cancer cells, and it can act as a prognostic predictor for patients with colorectal cancer (225). In summary, all four NDRG family members exhibit pro- or anti-cancer activity across various cancer types. A deep understanding of the complex and different roles of these members may be helpful in further understanding and treating cancer.

### The NDRG family and metastasis

#### NDRG family members regulate the epithelial-to-mesenchymal transition

EMT is a highly dynamic biological process that involves the functional transition of polarized epithelial cells into migratory, invasive, and apoptosis-resistant cells with a mesenchymal phenotype (226). While the EMT is required for normal embryonic development, tissue regeneration, organ fibrosis, and wound healing (226), it is a central initial step for cancer metastasis (227).

Our laboratory demonstrated that both the genetic or pharmacological induction of NDRG1 inhibited the TGF-β-induced EMT in HT29 and DU145 cells (11). Overexpression of NDRG1 inhibits canonical TGF-β signaling *via* its ability to suppress the expression and the subsequent translocation of Smad2/3, which decreases the expression of the E-cadherin repressor proteins, SNAIL and SLUG (11).

**Figure 6:**
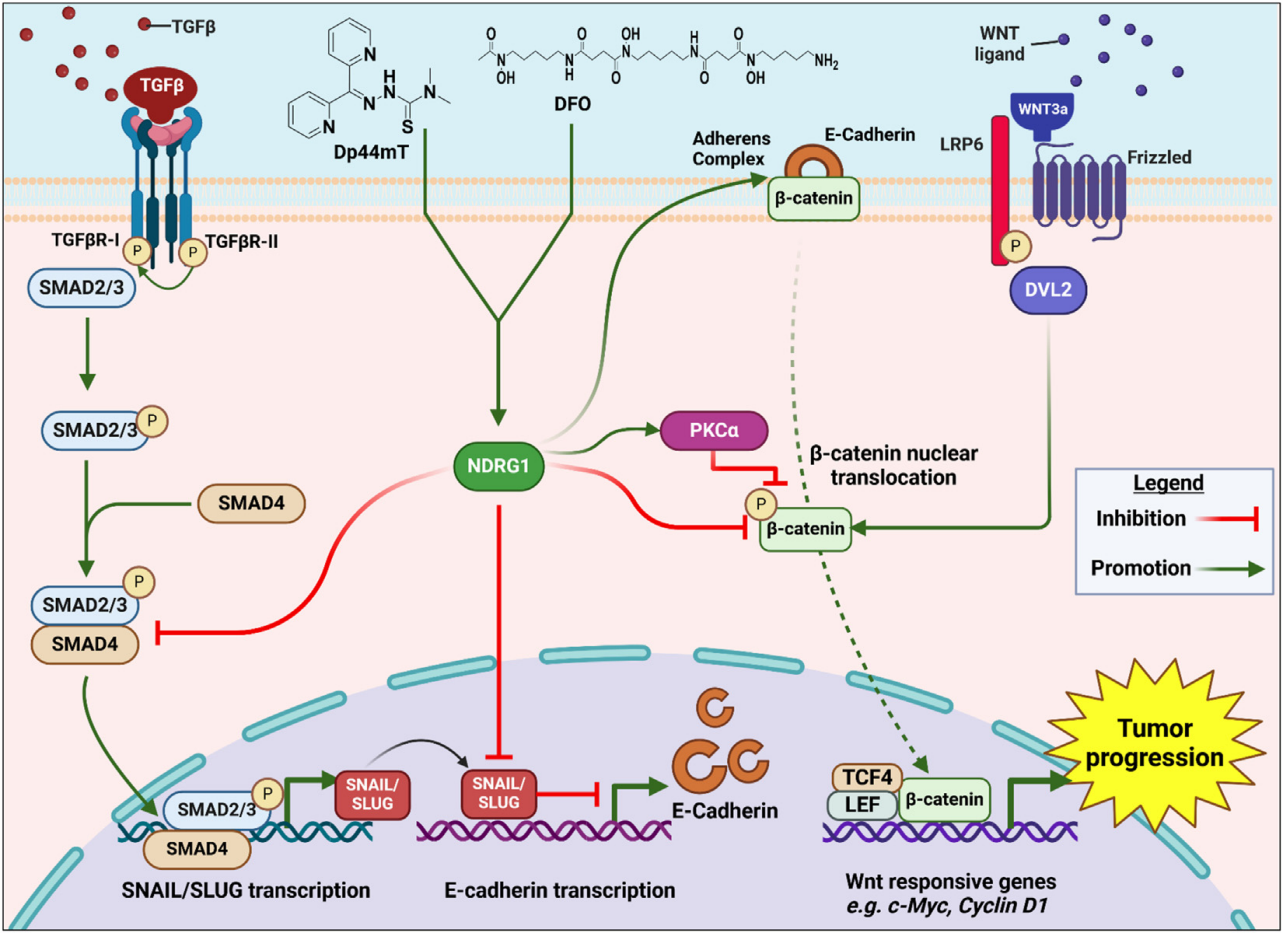
**Schematic demonstrating the role of NDRG1 in inhibiting the epithelial-mesenchymal transition and tumor progression *via* regulation of the TGF-β/SMAD and WNT pathways.** Iron-binding agents such as Dp44mT and DFO up-regulate the metastasis suppressor, NDRG1, to inhibit the TGF-β/SMAD pathway, which initiates the epithelial–mesenchymal transition (11). Increased NDRG1 levels result in decreased expression of SMAD2 and p-SMAD3 levels, preventing the association with SMAD4, which inhibits nuclear translocation. This decreased nuclear translocation of SMAD2, p-SMAD3, and SMAD4 decreases the TGF-β-induced expression of the transcriptional repressors, SNAIL and SLUG, which are responsible for *E-cadherin* transcriptional repression (11). Increased NDRG1 expression increases plasma membrane E-cadherin and β-catenin levels, that are involved in adherens complex formation at the plasma membrane, which suppress the initial step in metastasis mediated by the epithelial-mesenchymal transition (11). NDRG1 expression also negatively-regulates total β-catenin levels *via* PKCα (123). These events prevent β-catenin nuclear translocation, thus inhibiting the activation of the WNT signaling pathway and the transcription of *cyclin D1*, which plays an important role in cell cycle progression (122). Created in BioRender (Azad, M. (2025) https://BioRender.com/d74s214).

E-cadherin is a key protein in the adherens complex, as in the presence of calcium, its extracellular domain forms a homodimer with E-cadherin of neighboring cells, with its cytoplasmic domain interacting with α, β, and γ-catenins (228). Deletion or silencing of *CDH1* (which encodes E-cadherin) is prevalent in prostate, bladder, kidney, breast, stomach, and colon cancers (229, 230). Significantly, NDRG1 expression was demonstrated to facilitate the expression of E-cadherin and β-catenin at the plasma membrane, which is essential for adherens complex formation and is important for cellular adhesion that blocks migration (11) (Fig. 6). In contrast to its overexpression, *NDRG1* silencing increased migration and invasion of HT29 and DU145 cells and mimicked the EMT (11).

In concordance with these observations, NDRG1 is known to regulate the recycling of E-cadherin in cells by acting as an effector protein for Rab4a (231), This constituted part of a more general proposal that NDRG1 plays a role in vesicular trafficking and endosomal recycling. In fact, endosomal recycling studies indicated that NDRG1 co-localizes with transferrin that binds to the transferrin receptor 1, which is endocytosed *via* receptor-mediated endocytosis (231). These studies demonstrated that NDRG1 expression altered the kinetics of transferrin recycling in cells with NDRG1 knockdown, demonstrating a delay in transferrin recycling, while NDRG1 overexpression resulted in the opposite. Additionally, immunohistochemical analysis of a number of different human tissues demonstrated that NDRG1 was not only localized to the nucleus and cytoplasm but also in proximity to adherens junctions, where it was hypothesized to play a role in their stability (94).

In pancreatic cancer cell types, *NDRG1* silencing induces translocation of membrane E-cadherin and β-catenin into nuclei, leading to the activation of the pro-oncogenic WNT pathway, which includes cyclin D1, which promotes cell cycle progression and proliferation (210). NDRG1 expression induced, genetically or pharmacologically, facilitated the formation of E-cadherin/β-catenin complex on the plasma membrane and inhibited β-catenin nuclear translocation (210) (Fig. 6). This effect of NDRG1 prevented β-catenin acting as a pro-oncogenic transcription factor that induces *cyclin D1* transcription (210, 232). Therefore, NDRG1 inhibits the EMT in cancer cells *by* downregulating the TGF-β/Smad and WNT/β-catenin signaling pathways (210) (Fig. 6).

Emerging evidence has also demonstrated that NDRG2 acts as a tumor suppressor and is involved in regulating proliferation, differentiation, apoptosis, and metastasis in various malignant tumors (233, 234, 235). This study uncovered that metastatic tumor cells with a mesenchymal phenotype largely depend on glutamine utilization, with the gain of NDRG2 function using lentiviral expression abolishing the epithelial-mesenchymal transition and glutaminolysis in metastatic mucoepidermoid cancer (MC3) cells (235). Overexpression of NDRG2 was also responsible for suppressing the expression of the glutamine transporter, alanine-serine-cysteine transporter 2 (ASCT2), which leads to inhibition of glutaminolysis (235). This function occurred *via* the NDRG2-mediated suppression of c-Myc stabilization (235). AKT activation is known to stabilize c-Myc *via* phosphorylation and inactivation of GSK3β (236). GSK3β facilitates phosphorylation of c-Myc, which promotes its ubiquitination and degradation by Fbw7 (237). NDRG2 blocks AKT activity and conversely activates GSK3β, facilitating c-Myc downregulation (238). This downregulation of c-Myc represses ASCT2 expression and glutaminolysis in metastatic cancer cells with a mesenchymal phenotype.

Similarly, as observed for NDRG1 overexpression inhibiting the expression of the *E-cadherin* transcriptional repressor, Slug (13), subsequent studies using prostate cancer cells indicated NDRG2 overexpression decreased expression of another *E-cadherin* repressor, Snail (239). NDRG2 overexpression also inhibited metastases in the lungs of nude mice (235). GSK3β phosphorylation and activation by NDRG2 downregulate β-catenin in colorectal cancer (SW620) cells (58). NDRG2 interacts with E-cadherin and β-catenin in human colon adenocarcinoma cells either directly or *via* involvement of another intermediate molecule (58, 240).

Similar to the studies described above in prostate cancer cells (239), NDRG2 positively regulates the expression of E-cadherin *via* suppressing levels of the repressor, Snail, in several colon cancer cell-types and colon cancer tissues (241) (Fig. 6). This is important as the E-cadherin/β-catenin complex on the cell membrane forms a key component of the adherens complex, which maintains normal epithelial integrity (242). Disruption of this complex detrimentally affects cell adherence, which can induce cancer cell metastasis (243), as nuclear β-catenin translocation activates oncogenic WNT signaling in tumor cells (244). As such, NDRG1 and NDRG2 demonstrate functional similarities by promoting both E-cadherin and β-catenin expression at the plasma membrane to inhibit the EMT (11, 235) (Fig. 6).

NDRG3 is known to be involved in cellular proliferation and differentiation (31). Unlike NDRG1 and NDRG2, NDRG3 facilitates tumor progression and metastasis *via* activating the WNT/β-catenin pathway (245). Further, NDRG3 overexpression promotes proliferation and migration in prostate cancer (246). Overexpression of NDRG3 is associated with poorer overall survival in patients with invasive breast cancer (220), with it promoting metastasis by inducing Src phosphorylation in colorectal cancer (247). On the other hand, NDRG4 plays a tumor suppressive role in glioblastoma (248) and colorectal cancer (225).

### NDRG family members: Key players in multidrug resistance and sensitivity

Despite rapid advances in cancer treatment, significant mortality in patients with cancer is attributed to drug resistance (249). This can be related to multiple factors, including the enhanced expression and or activity of drug transporters, increased metabolism of chemotherapeutics, elevated production of growth factors such as vascular endothelial growth factor (VEGF), genetic factors, and elevated DNA repair (250). Recent studies have shown an association between NDRG family members and drug resistance in cancer (22, 251, 252, 253, 254). Conversely, other investigations have highlighted the association between NDRG proteins and drug sensitivity (22, 183, 254, 255). In particular, NDRG1’s role in both apoptotic and anti-apoptotic mechanisms in cancer has been widely explored (29, 162, 163, 180, 256, 257, 258, 259, 260).

#### NDRG1: emerging roles in multi-drug resistance

A number of investigations have examined the role of NDRG1 in multidrug resistance within multiple tumors (22, 251, 252, 253, 254). Zhang and colleagues demonstrated that NDRG1 overexpression in KP-N-NS neuroblastoma cells resulted in a significant increase in cell viability relative to control cells in response to incubation with vincristine, cisplatin, teniposide, and epirubicin (251). Furthermore, TUNEL assays also demonstrated decreased apoptosis under these conditions, suggesting drug resistance was increased *via* NDRG1 overexpression (251). In addition, proteins involved in the drug resistance phenotype, including P-glycoprotein (Pgp; ABCB1), low-density lipoprotein receptor-related protein 1 (LRP1), and multidrug resistance-associated protein 1 (MRP1; ABCC1), were also increased in NDRG1 overexpressing cells, further suggesting an NDRG1-mediated role in multidrug resistance of neuroblastoma (251).

Current standard-of-care therapies for glioma include radiotherapy and the alkylating chemotherapeutic temozolomide (TMZ) (261). Of note, *O*^6^-methylguanine-DNA methyltransferase (MGMT) is instrumental in the resistance of cells to alkylating chemotherapy *via* its role in DNA repair (262, 263, 264). Considering the role of hypoxia in inducing resistance to anti-cancer agents, Weiler *et al.* (264) hypothesized that NDRG1 may mediate this hypoxia-induced resistance to TMZ by associating with methyltransferases such as MGMT. In this study, *NDRG1* knockdown in human glioma cell lines resulted in sensitization to TMZ, and NDRG1 overexpression decreased TMZ-induced G_2_/M arrest (264). These observations suggest NDRG1 mediates hypoxia-induced resistance to alkylating agents.

**Figure 7:**
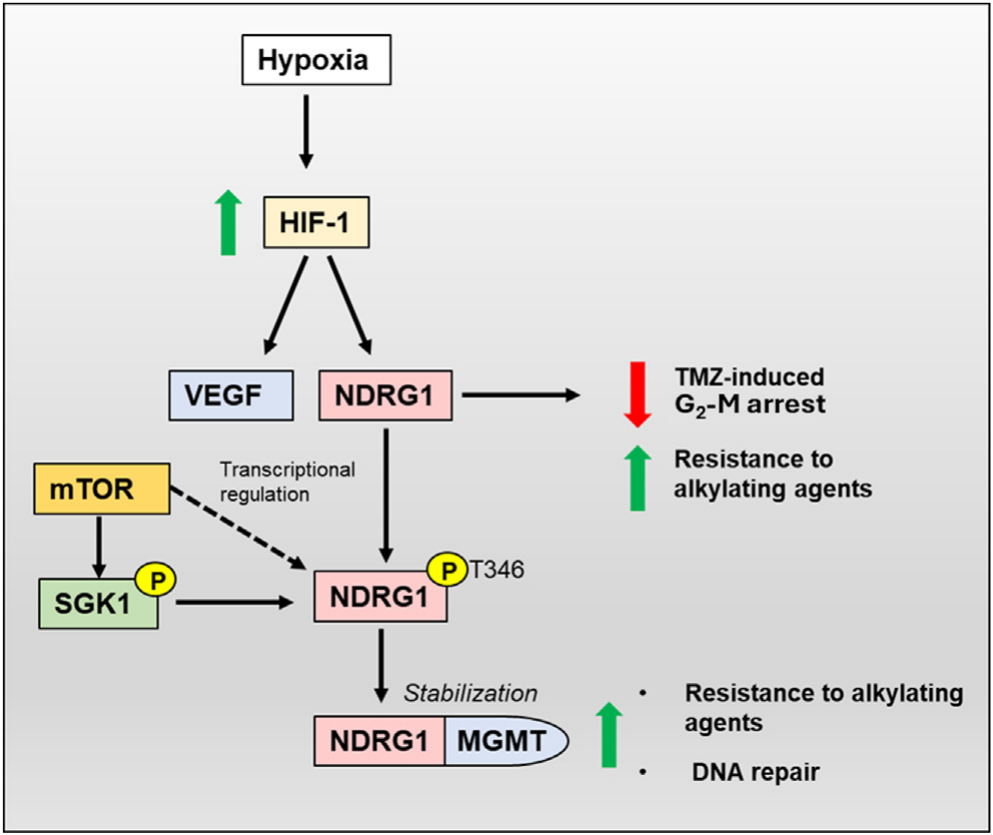
**Hypoxia-induced NDRG1-mediated resistance to alkylating chemotherapies such as Temozolomide (TMZ).** Under hypoxic conditions, increased HIF1α expression is induced that has been associated with resistance to alkylating agents. HIF-1 transcriptionally upregulates the expression of genes with hypoxia-response elements (HREs) including *vascular endothelial growth factor* (*VEGF*) and *NDRG1*. It has been demonstrated that hypoxia-induced NDRG1 expression results in a decrease in Temozolomide (TMZ)-induced G_2_/M arrest and resistance to TMZ (252). NDRG1 is also up-regulated by the mTOR downstream effector, SGK1, which phosphorylates and activates NDRG1 at Tyr 346 (301). SGK1 has also been shown to promote the direct association and stabilization of NDRG1 with MGMT, resulting in elevated DNA repair and resistance to TMZ (252).

It was also demonstrated that dexamethasone (DEX), a steroid administered as standard of care in glioma treatment, induced *SGK1* transcription resulting in increased phosphorylation of NDRG1 at T346 (Fig. 7) (252). The pharmacological inhibition of SGK1 using the compound, EMD638683, prevented the NDRG1-mediated protection of glioma from TMZ (252). This later pharmacological effect was not specific for glioma, as EMD638683 also decreased NDRG1 levels in pancreatic, breast, colon, and ovarian cancer cells (252). Of note, it was discovered *via* biomolecular fluorescence complementation assays that NDRG1 and MGMT directly associate in glioma cells (Fig. 6) (252). It was also observed that this critical interaction depended on SGK1 activity and that forced NDRG1 expression did not induce TMZ resistance upon *MGMT* knockdown (252). These data collectively suggest SGK1 plays a prominent role in NDRG1 activation and its association with and stabilization of MGMT, which promotes NDRG1-mediated TMZ resistance (Fig. 7).

Other NDRG family members have also been associated with drug resistance (102, 222). A recent study demonstrated that NDRG3 regulates imatinib resistance by promoting nuclear β-catenin accumulation in chronic myelogenous leukemia (CML) (222). High levels of NDRG3 were measured in patients with CML, and it was suggested that increased NDRG3 levels in K562 CML cells resulted in increased nuclear β-catenin accumulation and the subsequent upregulation of pro-oncogenic c-Myc and MDR1 (222).

Similar to NDRG1, NDRG2 has also been associated with drug resistance and p53-mediated apoptosis (102). Wei and colleagues (102) demonstrate that NDRG2 expression promotes sensitivity to the drug, adriamycin, in breast cancer cells by inhibiting proliferation in a p53-dependent manner (102). This is supported by data demonstrating that NDRG2 upregulates Bax expression by enhancing its half-life (102). Conversely, in HeLa cervical carcinoma cells, the knockdown of *NDRG2* was shown to promote cisplatin-induced apoptosis *via* the downregulation of BCL-2 (265). Although the role of NDRG2 in cisplatin sensitivity remains elusive, other reports indicate that NDRG2 increases cisplatin sensitivity *via* the modulation of the BAK to MCL-1 ratio in the NADPH oxidase-ROS-protein kinase R pathway (266).

### The NDRG family and their involvement in neurodegenerative diseases

Charcot-Marie-Tooth disease type 4D is a belligerent demyelinating peripheral neuropathy caused by autosomal recessive *NDRG1* null mutations (60). The neuropathology observed in this latter disease is thought to be induced by dysregulated lipid metabolism in Schwann cells, which play a critical role in the myelination of neurons in the peripheral nervous system (61). Considering this, NDRG family members have also been found to play an important role in neurodegenerative diseases, such as Parkinson’s disease and Alzheimer’s disease (44, 51, 52, 53, 54). Both RNA and protein expression of *NDRG2* are markedly upregulated in the hippocampi of patients with AD compared with healthy controls (267). Additionally, the expression of *NDRG2* was localized to cortical pyramidal neurons, dystrophic neurons, and senile plaques, which are all affected in AD (267). A similar upregulation of NDRG2 was also observed in a genetically induced rat model of AD (268). Both NDRG3 and NDRG4 expression were decreased in AD patient brains relative to the human normal adult brain (16, 269).

NDRG2 is highly expressed in astrocytes of the central nervous system and was also demonstrated to be expressed in the brains of humans with Parkinson’s disease and corticobasal degeneration (39). These results indicate that NDRG2 might play a role in the neurodegenerative changes of Parkinson’s disease. In addition, Herskowitz *et al.* (270) demonstrated increased NDRG2 phosphorylation in frontotemporal lobar degeneration. This latter result might suggest that apart from NDRG2 expression, NDRG2 phosphorylation is also modified during neurodegeneration.

NDRG2 has also been demonstrated to be upregulated in affected brain tissues and lesions from subjects suffering from confirmed late-onset AD and may play a role in the pathogenesis of this condition through several mechanisms (51, 267). Rong and associates (2017) discovered that using the human neuroblastoma cell line, SK-N-SH, NDRG2 modulates amyloid precursor protein (APP) processing, leading to an increase of amyloid β (Aβ) peptides *via* the BACE1 and GGA3 pathways (51).

These latter researchers also found in human neuroblastoma cells that the phosphorylation of tau, another hallmark of AD, was decreased after *NDRG2* knockdown (51). Conversely, this group also demonstrated that *NDRG2* overexpression in these cells facilitated tau phosphorylation (51), a process widely considered a pathological hallmark of AD (271). Investigations also demonstrated possible neuroprotective roles of NDRG2, as demonstrated by the ability of NDRG2 expression in male mice to prevent ischemic brain edema (272). In contrast, knockout of *NDRG2* in mouse brains resulted in increased susceptibility to stroke-induced brain edema and astrocytic swelling *via* an imbalance in Na^+^ and water transfer, suggesting a role of NDRG2 in electrolyte homeostasis (272).

On the contrary, the expression of the other two NDRG family members, NDRG3 and NDRG4, were lower in the brains of patients with Alzheimer’s disease relative to human normal adult brain (16, 269). In Parkinson’s disease, NDRG1 expression was demonstrated to be high in astrocytes in the cerebral cortex of adult 57BL/6J mice (52). An investigation by Tacheichi *et al.* (2011) demonstrated that NDRG2 may be involved in the regulation of astroglial activation *via* suppression of cellular proliferation (39). The progression of Parkinson’s disease has previously been reported to be related to the *Parkin* gene mutation (273). By analyzing gene expression profiles of patients with Parkinson’s disease from the Gene Expression Omnibus database, 11 genes involved in the initiation stage of Parkinson’s disease were identified. This study by Wang *et al.* identified transcription factors, junction plakoglobin and NDRG1 were regulated by miR-133 (274), a miRNA which has been highly implicated in Parkinson’s disease (275).

In contrast to the other members of the NDRG family, NDRG4 may be negatively associated with Parkinson’s disease (276). The gene *NDRG4* has a lower expression in the cingulate gyrus and substantia nigra of patients with Parkinson’s disease relative to healthy controls (276). A recent study analyzed DNA methylation data and identified significant DNA methylation positions (DMPs) near the *NDRG4* gene, and 4 hyper-methylated cytosine-phosphate-guanine (CpG) regions (276). This latter study identified six genes (including *NDRG4*) demonstrating differential methylation and expression in the cingulate gyrus, suggesting that changes in DNA methylation of these genes may be affecting their expression. Interestingly, *NDRG4* was also shown to be differentially methylated and expressed in the substantia nigra, an area of the brain associated with Parkinson’s disease (276).

## NDRG family members as therapeutic targets for cancer treatment

### Targeting NDRG1 as a novel therapy against metastasis

**Figure 8:**
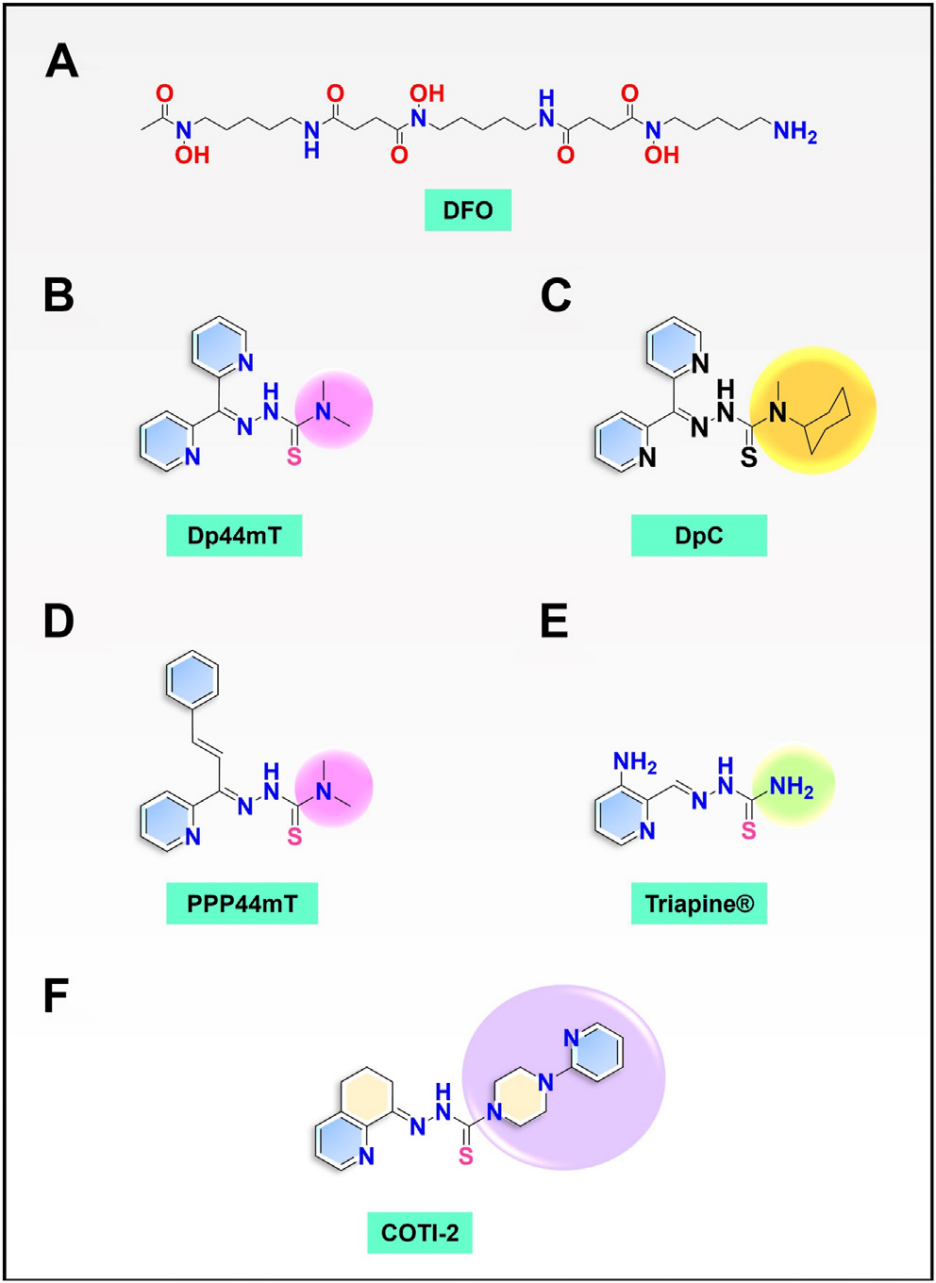
**The line drawings of the chemical structures of the ligands.***A*, Desferrioxamine (DFO); *B*, Dp44mT; *C*, DpC; *D*, PPP44mT; *E*, Triapine; and *F*, COTI-2.

Iron is essential for cancer cell proliferation due to its critical role in driving key pathways such as DNA synthesis, mitochondrial respiration, and redox regulation (277). Iron-binding ligands such as desferrioxamine (DFO; Fig. 8*A*), and the thiosemicarbazone ligands, Dp44mT (Fig. 8*B*), DpC (Fig. 8*C*), and (*E*)-3-phenyl-1-(2-pyridinyl)-2-propen-1-one 4,4-dimethyl-3-thiosemicarbazone (PPP44mT) (Fig. 8*D*) demonstrate anti-proliferative activity against a range of tumor models *in vitro* and *in vivo* (278). In fact, some of these agents (*i.e.*, DFO and DpC) and other thiosemicarbazone ligands, such as 3-aminopyridine-2-carboxaldehyde thiosemicarbazone (Triapine; Fig. 8*E*) and (*E*)-*N*′-(6,7-dihydroquinolin-8(5H)-ylidene)-4-(pyridin-2-yl)piperazine-1-carbothiohydrazide (COTI-2; Fig. 8*F*), have entered clinical trials for cancer treatment (279). The high iron requirement by cancer cells makes them highly susceptible to iron depletion *via* iron-binding ligands (280). One of the key molecular targets of DFO, Dp44mT, and DpC is the metastasis suppressor, NDRG1 (29). Previous research has demonstrated that cancer cell iron depletion by DFO, Dp44mT, and multiple other ligands significantly upregulated NDRG1 and is correlated with the iron-chelating efficacy and anti-proliferative activity of these agents (134, 144). However, iron chelators can affect multiple iron-dependent enzymes and signaling pathways. Thus, the anti-cancer activity of these agents extends beyond NDRG1 induction alone (11, 12, 13, 83, 87, 111, 128, 132, 180, 210, 281, 282).

Iron chelating agents such as DFO, Dp44mT, and DpC upregulate NDRG1 expression *via* HIF-1α-dependent and -independent mechanisms (134, 144). Furthermore, HIF-1α-independent upregulation of NDRG1 can also be induced by tumor cell iron depletion (134, 144). Cellular iron depletion also induces HIF1α-independent induces eukaryotic initiation factor 3a (eIF3a)-positive stress granules and decreases eIF3a expression, with these low eIF3a levels prioritizing translation of NDRG1 mRNA transcripts (134, 144). Moreover, multiple studies have demonstrated that p53 expression is upregulated by cellular iron chelation (283, 284, 285, 286), an upstream transcriptional regulator of *NDRG1* (162).

The mechanism by which iron-binding ligands prevent metastasis *via* NDRG1 appears to be intricate and multifaceted. However, inhibition of the TGF-β-induced epithelial-mesenchymal transition *via* the overexpression of NDRG1 induced genetically or by the iron-binding ligands, DFO or Dp44mT, could play a key role in suppressing metastasis (11). These later studies indicate the importance of NDRG1 as a major molecular target of iron ligation (11). However, the lead compounds from the first and second generations of di-2-pyridyl ketone thiosemicarbazones, namely Dp44mT and DpC, potently upregulate NDRG1, but unfortunately, they demonstrated off-target effects such as the detrimental oxidation of oxy-hemoglobin and oxy-myoglobin (281). This effect was thought to lead to muscle pain after the treatment of patients with orally administered DpC and resulted in the discontinuation of the clinical trial (279).

To overcome the issue of oxidizing heme in key oxygen-carrying hemoproteins, a new series of ligands was recently designed and developed, namely those of the 3-phenyl-1-(2-pyridinyl)-2-propen-1-one-thiosemicarbazone (PPPT) series (278). The lead compound, PPP44mT, showed distinctive properties mediated by the inclusion of a styrene moiety. Through steric and electrochemical mechanisms, PPP44mT markedly suppressed deleterious oxy-myoglobin or oxy-hemoglobin oxidation relative to other potent thiosemicarbazones, namely, Dp44mT and DpC, but maintained the ability to significantly upregulate NDRG1 (278).

Further, PPP44mT demonstrated potent and selective anti-proliferative activity against neuroepithelioma (an aggressive brain tumor cell-type) and pancreatic cancer cells, and suppressed WNT signaling, resulting in a pronounced decrease in cyclin D1 levels (278). This agent also inhibited the expression of key pro-oncogenic signaling proteins and their activation, including EGFR, p-EGFR (Tyr1045), p-c-Src (Tyr416), and PI3K p-p85 (278), while upregulating PKCα that plays a role in decreasing WNT signaling by phosphorylating β-catenin at Ser33, Ser37, and Thr41 (278). In fact, the pharmacological upregulation of NDRG1 by PPP44mT and other NDRG1-inducing agents (*i.e.*, DFO, Dp44mT, DpC) mimicked the effect of genetic NDRG1 overexpression on PKCα levels and its downstream effectors (123).

While the third-generation PPPT analogs such as PPP44mT could markedly suppress oxidation of oxy-Mb generation than their first and second-generation counterparts (*e.g.*, Dp44mT and DpC), they could not completely prevent it (278). As such, the development of NDRG1-inducing thiosemicarbazones that totally inhibit oxy-Mb oxidation and also maintain NDRG1 upregulation were important to design and develop (287). The novel recently synthesized thiosemicarbazone, 1-(pyridin-2-yl)-3-(p-tolyl)prop-2-en-1-one-4-cyclohexyl-4-methyl-3-thiosemicarbazone (PPTP4c4mT) and its selenium analog, totally prevented oxy-Mb oxidation, upregulated NDRG1, and downregulated multiple key receptors that display inter-receptor cooperation leading to aggressive and resistant breast cancer (287). In fact, both these new agents demonstrated similar activity to the clinically trialed agents, DpC, Triapine, and COTI-2, at downregulating EGFR, estrogen receptor-α (ER-α), AR, progesterone receptor (PR), prolactin receptor (PRLR), and cyclin D1, and upregulating NDRG1 expression (287). Notably, *NDRG1* silencing prevented the ability of the first-generation thiosemicarbazone, Dp44mT, to inhibit metastasis *in vivo* in a human breast cancer tumor model (7). These latter results indicate the anti-metastatic activity of Dp44mT relates to its ability to upregulate NDRG1 in breast cancer cells.

In addition to suppressing metastasis, NDRG1 expression has been demonstrated to inhibit bi-directional signaling between pancreatic cancer cells and pancreatic stellate cells (282, 288). This signaling between these two cell types is important for the promotion of pancreatic cancer growth and the generation of desmoplasia (282, 288). Desmoplasia refers to the dense stromal fibrosis observed in pancreatic cancer tumors that is generated by activated pancreatic stellate cells and retards the entrance of chemotherapy into the tumors. In fact, activated pancreatic stellate cells synthesize and release hepatocyte growth factor (HGF) and insulin-like growth factor (IGF-1) that promote tumorigenesis and resistance (282, 288).

Studies from our laboratory demonstrated that genetic NDRG1 overexpression inhibits pancreatic cancer-pancreatic stellate cell crosstalk that inhibits HGF and IGF-1 signaling between stellate cells and tumor cells and suppresses sonic hedgehog signaling (282, 288). The NDRG1-inducing thiosemicarbazone, DpC, inhibited tumor growth, metastasis, and desmoplasia to a markedly greater extent than standard chemotherapy, namely gemcitabine. While DpC inhibited pancreatic cancer cell growth, it also reprogrammed stellate cells to an inactive state, inhibiting their collagen synthesis, which is a critical component of the desmoplastic reaction (282, 288). These studies demonstrated the role of targeting NDRG1 in suppressing oncogenic bidirectional signaling between stellate cells and tumor cells to overcome key pathogenic mechanisms in pancreatic cancer.

Further studies examining pancreatic cancer cells and stellate cells then examined the effect of NDRG1 and DpC on the protein ligands, WNT3a and tenascin C (TnC), that are also secreted by stellate cells that subsequently act on PC cells, leading to pro-oncogenic β-catenin and YAP/TAZ signaling (282, 288). Pharmacological induction of NDRG1, implementing DpC, inhibited WNT/TnC-mediated interactions between PC cells and stellate cells. This effect was mediated by suppression of WNT3a and TnC secretion by stellate cells and attenuation of WNT/β-catenin and YAP/TAZ activation and signaling in pancreatic cancer cells (282, 288). In fact, inhibition of β-catenin and YAP/TAZ nuclear localization was observed together with upregulation of the WNT pathway inhibitor, DKK1.

The expression of NDRG1 also suppressed TGFβ secretion by pancreatic tumor cells, which activates pancreatic stellate cells (13). In an orthotopic pancreatic cancer mouse model, treatment with the NDRG1-inducing agent, DpC (10 mg/kg, 3 × week), *via* oral gavage decreased the expression of β-catenin, TnC, and YAP/TAZ compared to the vehicle control (13, 288). The decrease in YAP/TAZ levels in the pancreatic cancer xenografts after treatment with DpC was greater than that compared with the standard chemotherapeutic drug, Gemcitabine (13). These studies demonstrated that both genetically overexpressed NDRG1 and pharmacologically induced NDRG1 inhibit WNT/TnC-mediated paracrine interactions between pancreatic tumor cells and stellate cells. In summary, the pro-oncogenic bidirectional signaling between pancreatic cancer and stellate cells can be pharmacologically targeted through the upregulation of NDRG1.

### Targeting NDRG2 as a novel therapy against metastasis

As previously demonstrated with NDRG1 (134), NDRG2 is similarly regulated *via* a HIF-1α-dependent mechanism *via* the iron-binding ligand, Dp44mT (289, 290). A study by Zhou and colleagues demonstrated that Dp44mT can increase *NDRG2* transcription *via* retinoic acid receptor-related orphan receptor-α (RORA) (289, 290). Furthermore, the knockdown or overexpression of RORA resulted in down- and upregulation of NDRG2, respectively, supporting the association between Dp44mT, RORA, and NDRG2 expression (290, 291). Other studies demonstrated that hypoxia regulates *RORA* and that it is a HIF-1 target gene (292). Collectively, these investigations suggest RORA-mediated upregulation of NDRG2 by a HIF-1-dependent mechanism (290, 291, 292, 293).

Analogously to *NDRG1*, *NDRG2* is also known to be a target gene of the tumor suppressor, p53 (291). As such, the known upregulation of p53 caused by cellular iron deprivation (283) could potentially lead to the NDRG2 expression. A study by Wang *et al.* 2014 demonstrated that Dp44mT inhibited the metastasis of hepatocellular carcinoma cells by upregulating NDRG2, which activates the gp130/STAT3 pathway (289). This study suggested the decreased expression of gp130 and the negative regulation of STAT3 signaling by NDRG2 overexpression was responsible for the Dp44mT-mediated inhibition of the EMT and apoptosis *via* decreasing TGF-β/SMAD signaling (289). Of relevance, Oh *et al.* (2012) and Lee *et al.* (2008) demonstrated that overexpression of NDRG2 is involved in attenuating TGF-β-mediated tumor cell invasion by decreasing TGF-β synthesis (294, 295).

### Targeting NDRG3 and NDRG4 as a novel therapy against metastasis

There are limited studies on the mechanism of action of iron-binding ligands on the regulation of NDRG3 and NDRG4. Lee *et al.* (2016) demonstrated that the iron-binding ligands, DFO, deferasirox, and Dp44mT, upregulated the expression of NDRG1 and NDRG3, while having no significant effect on NDRG2, and inhibited cell cycle progression and the proliferation of oral squamous cell carcinoma cells (14). In fact, further studies by these authors demonstrated that *NDRG3* knockdown in an oral squamous carcinoma cancer cell line (OECM-1) exhibited significantly higher ^3^H-thymidine incorporation *in vitro*, indicating higher DNA synthesis, and greater tumor xenograft growth *in vivo* in nude mice (14). These studies support a suppressive role of NDRG3 expression in this cell type.

These results described above are somewhat contrary to other studies demonstrating NDRG3 overexpression enhances tumor cell proliferation and metastasis in multiple cancer types, including hepatocellular carcinoma, prostate, colorectal, and breast cancer (296). However, the exact mechanism of how iron-binding agents regulate NDRG3 expression in oral squamous carcinoma cells remains unclear (14), with the effects of these ligands being multifaceted as they bind iron that plays metabolically diverse roles (297, 298, 299). In fact, apart from the upregulation of NDRG1 and NDRG3 by the iron-binding ligands, cyclin D1 expression was also suppressed (14), which is a known target of this class of agents that inhibits growth (210, 232). Although the effect of iron-binding drugs on NDRG4 expression is yet to be explored, considering the tumor-suppressive role of this protein (144, 145), investigation of NDRG4 as a therapeutic target would be of interest.

## Perspective backs and conclusions

While extensive research has been undertaken to understand the activities of NDRG family members (21, 57, 201, 202, 251), there remain key areas that require further examination. Of all the members of the NDRG family, NDRG1 has garnered the most attention, with the roles and regulatory mechanisms of the remaining family members (NDRG2, NDRG3, and NDRG4) being unclear, especially in the context of coincident NDRG1 expression.

Emerging evidence highlighting the pro-oncogenic role of NDRG1 in specific cancer types indicates the need for further studies to understand the factors that dictate its pro-oncogenic *versus* tumor-suppressive roles (21, 57, 201, 202, 251). A better understanding of the determinants that switch NDRG1 from a tumor suppressor to a tumor promoter can lead to potential targeted anti-cancer therapies. Intriguing evidence of the involvement of NDRG family members in neurodegenerative diseases suggests novel roles and their potential as therapeutic targets (16, 44, 51, 52, 53, 54, 267, 269).

For future perspectives, ongoing research into the NDRG family members will increasingly utilize cutting-edge techniques, such as functional genomics using CRISPR and high-throughput interaction screening, to discover their molecular interactions, regulatory pathways, and potential roles in pathogenesis. This will illuminate the functions of specific protein motifs or domains, post-translational modifications, and protein-protein interactions in mediating NDRG-associated roles and disease phenotypes. This will also enable targeted therapeutic interventions and better patient outcome predictions. In addition, drug discovery efforts into the identification and optimization of NDRG-inducing drugs, particularly novel small molecules, are promising for clinical applications, particularly in oncology. Ultimately, the integration of NDRG family-based biomarkers into patient stratification approaches can enhance personalized therapeutic interventions, redefining the role of these proteins as targets for precision medicines, especially in cancer and neurodegenerative disease.

In conclusion, this review highlights the mechanisms of action and multifaceted role of the NDRG family members in diseases such as cancer and neurodegeneration, with NDRG1 being the most studied due to its well-characterized function as a metastasis suppressor. Although the roles of NDRG2, NDRG3, and NDRG4 have been explored less, there is emerging evidence that they may play different roles than NDRG1 regarding tumor suppression and neuronal function. The complex regulation of the NDRG family members *via* transcriptional regulation, post-translational modification, and association with other key proteins underscores their intricate molecular biology. The dualistic nature of NDRG1 family members and their function as tumor suppressors or pro-oncogenic proteins necessitates careful examination to pave the way for novel therapeutic interventions.

## Conflict of interest

The authors declare that they have no conflicts of interest with the contents of this article.
